# Fluorescent Thienothiophene-Containing Squaraine Dyes and Threaded Supramolecular Complexes with Tunable Wavelengths between 600–800 nm

**DOI:** 10.3390/molecules23092229

**Published:** 2018-09-01

**Authors:** Wenqi Liu, Hannah H. McGarraugh, Bradley D. Smith

**Affiliations:** Department of Chemistry and Biochemistry, 236 Nieuwland Science Hall, University of Notre Dame, Notre Dame, IN 46556, USA; wenqi.liu@northwestern.edu (W.L.); hmcgarra@nd.edu (H.H.M.)

**Keywords:** near infrared dye, fluorescence, supramolecular recognition, non-covalent, host guest, synthesis, quantum yield, multiplex imaging

## Abstract

A new family of fluorescent thiophene and thienothiophene-containing squaraine dyes is described with tunable wavelengths that cover the absorption/emission range of 600–800 nm. The deep-red and near-infrared fluorescent compounds were easily prepared by simple synthesis and purification methods. Spectral studies showed that each squaraine was rapidly encapsulated by a tetralactam macrocycle, with nanomolar affinity in water, to produce a threaded supramolecular complex with high chemical stability, increased fluorescence quantum yield, and decreased fluorescence quenching upon dye self-aggregation. Energy transfer within the supramolecular complex permitted multiplex emission. That is, two separate dyes with fluorescence emission bands that match the popular Cy5 and Cy7 channels, could be simultaneously excited with a beam of 375 nm light. A broad range of practical applications is envisioned in healthcare diagnostics, microscopy, molecular imaging, and fluorescence-guided surgery.

## 1. Introduction

Organic dyes that emit fluorescence in the near infrared (NIR) window (650–900 nm) are needed for biological imaging as this wavelength region has relatively deep tissue penetration, minimal tissue auto fluorescence, and lower Rayleigh scattering of the light [1,2,3]. At present, many NIR fluorescent probes are based on cyanine dyes because of their large molar absorption coefficients, moderate to high fluorescence quantum yields, and broad wavelength tunability [4]. Although the practical value and utility of cyanine dyes in biological studies is without doubt, they can exhibit some non-optimal molecular properties such as poor chemical and photostability, limited water solubility, propensity to self-quench upon dye aggregation, and difficult purifications due to the ionic molecular structures [5]. Thus, an ongoing task for dye chemists is to prepare new classes of NIR fluorescent dyes for biological imaging applications.

**Scheme 1:**
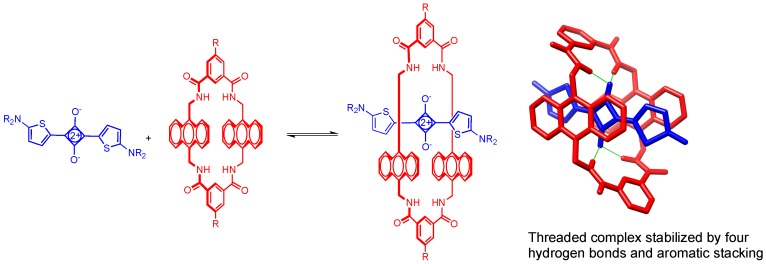
Encapsulation of a bis(aminothiophene)squaraine dye by a tetralactam macrocycle that has two anthracene sidewalls.

Squaraine dyes are a well-known family of NIR fluorescent dyes with intense and narrow absorption bands, high fluorescence quantum yields, and excellent photostabilities [6]. Because of these attractive optical properties, squaraines have been investigated over the years for many potential applications [7,8,9,10,11]. In the specific case of biological imaging, the development of squaraine dyes as NIR fluorescent probes has been limited by technical challenges such as poor chemical stability in biological media and a propensity for dye self-aggregation [12]. Our research group has discovered that both problems can be abrogated by encapsulation of the dye inside a protective macrocycle [13]. The equilibrium in Scheme 1 shows complexation of an bis(aminothiophene)squaraine dye by a tetralactam macrocycle that has two anthracene sidewalls. The threaded complex is stabilized by a synergistic combination of aromatic stacking and hydrogen bonding interactions between the two supramolecular components, and complexation produces a red-shift of the squaraine absorption/emission maxima [14]. Moreover, the surrounding macrocycle protects the encapsulated squaraine dye from nucleophilic attack, attenuates the quenching effects caused by dye self-aggregation, and enhances the squaraine quantum yield in water [14,15,16,17]. The favorable fluorescence properties of these threaded squaraine macrocycle complexes have been exploited for different applications in biological imaging [18,19], diagnostics [20] and liposome surface functionalization [21]. Most squaraine dyes and squaraine/macrocycle complexes (including the system in Scheme 1) have fluorescence emission bands in a narrow region between 650–700 nm, which makes them useful substitutes for the common cyanine dye, Cy-5, in many types of microscopy and in vivo imaging techniques [3,18]. Looking to the future, there is little doubt that the value of squaraine dyes for biological imaging would be enhanced if additional dye structures could be produced with absorption/emission wavelengths that cover a broader window and extend up to 850 nm. Even more utility would be gained if the squaraine structures allowed macrocycle threading to produce high stability complexes. Within the literature on squaraine dyes is a small and scattered collection of structures with squaraine absorption/emission bands that are close to 800 nm [22,23,24,25,26]. None of these were considered suitable for our needs because the structures were either too hard to prepare as water-soluble molecules or they contained sterically large terminal groups that would prevent macrocycle threading.

**Scheme 2:**
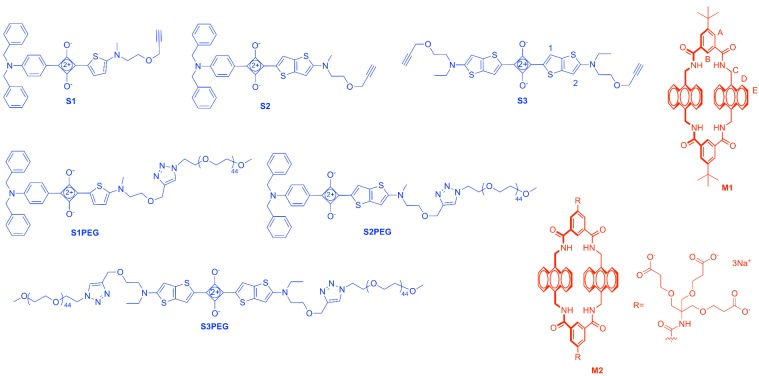
Structures of squaraine dyes and macrocycles used in this study with relevant atom labels.

We decided to develop a new set of squaraine chromophores with incrementally extended absorption/emission wavelengths. We knew that squaraine structures containing aminophenyl or aminothiophene units would be useful for operation in the region of 600–700 nm [27]. The real challenge was to develop a stable squaraine chromophore with the extended π-conjugation needed to reach 800 nm and also allow macrocycle threading. After some preliminary experimentation, we discovered that squaraine dyes could be prepared with attached aminothienothiophene units, and that these dyes exhibited the desired optical and supramolecular properties. Herein, in Scheme 2 we showcase this discovery by describing three organic-soluble dyes (**S1**–**S3**) that were easily prepared from commercially available materials in a few steps, and water-soluble analogues (**S1PEG**–**S3PEG**) that were obtained by appending long polyethylene glycol (PEG) chains. We find that the dyes form supramolecular complexes with the tetralactam macrocycles **M1** or **M2** to produce a suite of 12 fluorescent compounds that span the absorption window of 600–800 nm, making them a very attractive set of dyes for a wide range of future biological imaging applications.

## 2. Results and Discussion

### 2.1. Synthesis

**Scheme 3:**
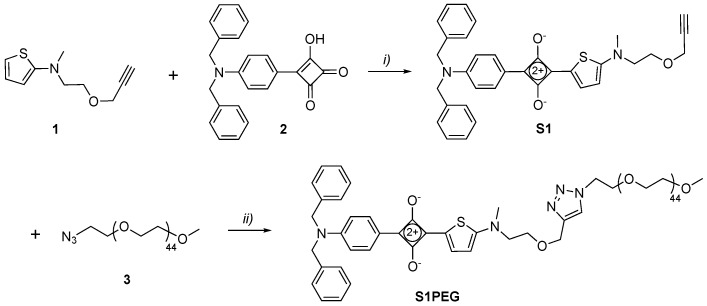
Reagents and conditions: (i) Benzene, 1-butanol, 100 °C, 2 h, 77%; (ii) Tris[(1-benzyl-1*H*-1,2,3-triazol-4-yl)methyl]amine (TBTA) Cu(I)Br, triethylamine, CHCl_3_, rt, 12 h, 92%.

**Scheme 4:**
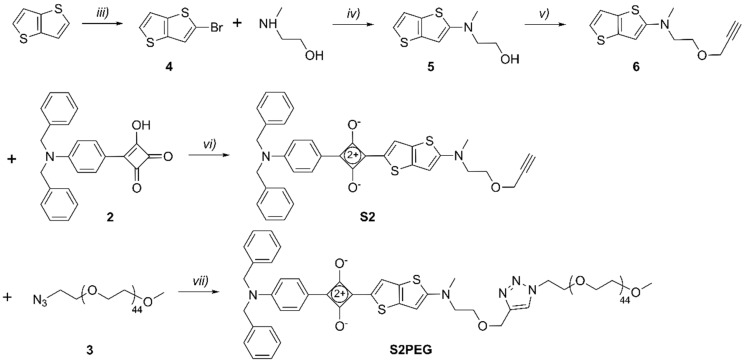
Reagents and conditions: (iii) *N*-bromosuccinimide, AcOH, 2h, rt, 81%; (iv) K_3_PO_4_·H_2_O, Cu^0^, CuI, 90 °C, 5 h, 46%; (v) Propargyl bromide, NaOH, toluene, tetrabutylammonium (TBA) HSO_4_, rt, 5 h, 97%; (vi) Benzene, 1-butanol, 100 °C, 2 h, 58%; (vii) TBTACu(I)Br, *N*,*N*-Diisopropylethylamine (DIPEA), CHCl_3_, rt, 12 h, 55%.

The unsymmetrical squaraines, **S1** and **S2,** were synthesized by similar procedures using *N*,*N*-dibenzylaminophenyl semisquaraine **2** as a common building block (Scheme 3 and Scheme 4). In the first case, condensation of **2** with 2-aminothiophene derivative **1** furnished organic-soluble squaraine **S1** in 77% yield, which was subsequently converted into water-soluble squaraine **S1PEG** by conducting a copper-catalyzed alkyne-azide cycloaddition reaction with **3**. In the second case, the appropriate 2-aminothienothiophene **6** was produced in three steps [28] and then condensed with **2** to give organic-soluble squaraine **S2** in 58% yield. A subsequent cycloaddition reaction with **3** provided the water-soluble squaraine **S2PEG**. 

**Scheme 5:**
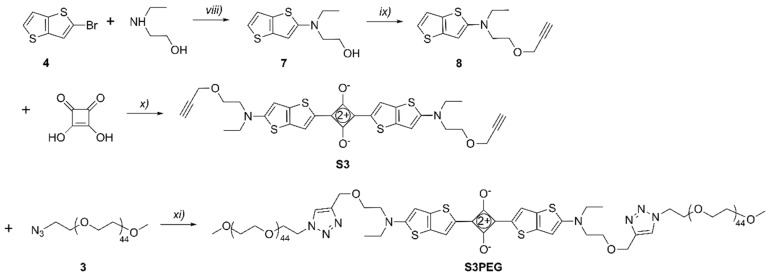
Reagents and conditions: (viii) K_3_PO_4_·H_2_O, Cu^0^, CuI, 90 °C, 5 h, 51%; (ix) Propargyl bromide, NaOH, toluene, TBA HSO_4_, rt, 5 h, 84%; (x) Benzene, 1-butanol, 100 °C, 2 h, 92%; (xi) TBTACu(I)Br, DIPEA, CHCl_3_, rt, sonication, 4 h, 42%.

The symmetrical squaraine **S3** was prepared (Scheme 5) in 92% yield by condensing squaric acid with two molar equivalents of 2-aminothienothiophene **8**. The reaction was complete after 2 hours and the air-stable product appeared as a golden brown precipitate that could be isolated by simple filtration. A subsequent cycloaddition reaction with two molar equivalents of **3** provided the water-soluble squaraine **S3PEG**.

### 2.2. Macrocycle Threading in Chloroform

**Figure 1:**
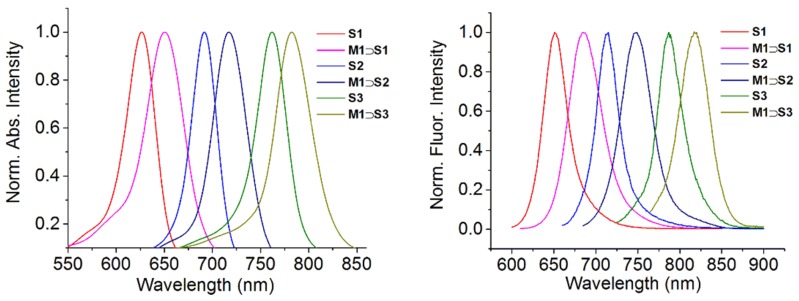
Absorption (**left**) and emission (**right**) maxima for separate solutions of **S1**, **S2** and **S3** and their threaded complexes with **M1** in CHCl_3_.

As a series, squaraines **S1**, **S2** and **S3** have increasing π-conjugation, and they are soluble in organic solvents such as CHCl_3_. A key molecular design feature is the small *N*-alkyl group (either *N*-methyl or *N*-ethyl) at one or both ends of the central chromophore, which ensures that macrocycle threading is facile [17]. Threading studies in CHCl_3_ were performed by mixing separate samples of **S1**, **S2** and **S3** with one molar equivalent of organic-soluble macrocycle **M1**. In each case there was an immediate 20–35 nm red-shift in the squaraine absorption and emission maxima wavelength (Figure 1) which is diagnostic of squaraine encapsulation by **M1**. 

**Figure 2:**
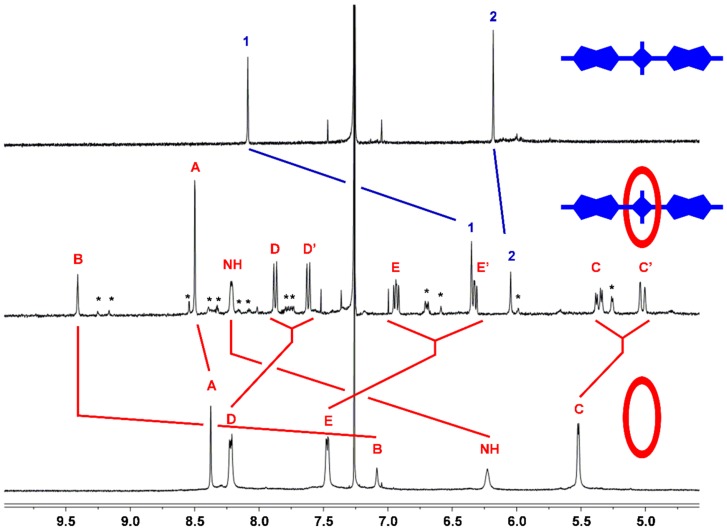
Comparison of partial ^1^H-NMR spectra for separate samples of free **S3**, **M1** ⊃ **S3**, and free **M1** in CDCl_3_. Atom labels are provided in Scheme 2. Red and blue lines indicate major changes in chemical shifts upon complexation. * designates NMR signals for the minor **M1** ⊃ **S3** complex with encapsulated **S3** in a cis conformation (trans/cis ratio for encapsulated **S3** is >10:1). See Scheme 2 for atom labels which are numbers (for **S3**) and letters (for **M1**).

**Figure 3:**
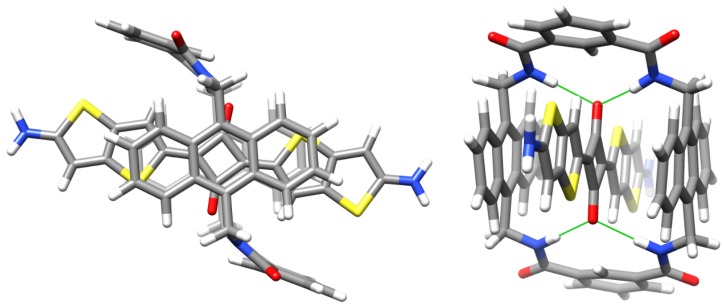
Molecular model of **M1** ⊃ **S3** (squaraine and macrocycle alkyl groups removed for clarity), calculated by the semiempirical method at PM7 level, showing the encapsulated squaraine in a trans conformation and ends of the squaraine’s thienothiophene units outside the shielding zone of **M1**. (**left**) front view, (**right**) side view. Hydrogen bonds between macrocycle NH residues and squaraine oxygen atoms are indicated by green lines. Color code for atoms: grey = carbon, blue = hydrogen, blue = nitrogen, red = oxygen, yellow = sulfur.

In the case of **S3**, the macrocycle threading process was characterized using ^1^H-NMR. In Figure 2 is a comparison of partial ^1^H-NMR spectra for separate solutions of **S3**, **M1** and **M1** ⊃ **S3** in CDCl_3_. Squaraine **S3** can adopt two low-energy conformations that differ in the relative orientation of the thienothiophene units, which can have a cis or a trans relationship. The ^1^H-NMR spectrum for free **S3** shows single peaks for protons 1 and 2 suggesting fast exchange between cis and trans conformations. A sample containing a 1:1 mixture of **S3** and **M1** instantly formed **M1** ⊃ **S3** in a quantitative yield. The chemical shifts for **M1** ⊃ **S3** indicated encapsulation of the squaraine inside the macrocycle as illustrated by the molecular model in Figure 3. Notably, there are large downfield changes in chemical shift for the macrocycle NH residues and protons B, and large upfield changes in the chemical shift for macrocycle protons D and E. Moreover, the spectral patterns for the macrocycle unambiguously indicate that the encapsulated squaraine predominantly adopts a C_2_-symmetric trans conformation, with a small fraction of the encapsulated squaraine (<10%) in a cis conformation. For example, the major signal for the macrocycle B protons is a singlet at 9.33 ppm, which can only arise if the encapsulated squaraine is trans. In contrast, the minor signal for the macrocycle B protons is split into two singlets around 9.22 ppm, which implies that the encapsulated squaraine is cis. The peaks for squaraine protons 1 and 2 in encapsulated **S3** are sharp due to hindered rotation of the squaraine single bonds when **S3** is inside the macrocycle. Moreover, the large upfield change in chemical shift for squaraine proton 1 and the relatively negligible change in chemical shift for proton 2 are both consistent with the anisotropic NMR shielding zones predicted by the molecular model in Figure 3.

### 2.3. Macrocycle Threading in Water

**Figure 4:**
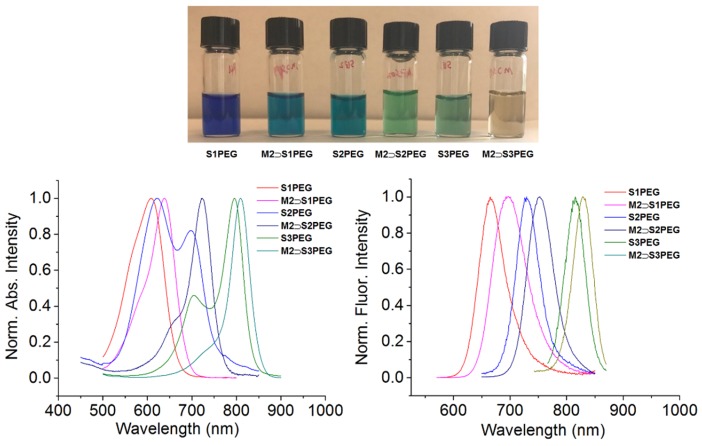
(**top**) Photograph of separate solutions containing free squaraine or macrocycle complex in H_2_O. Absorption (**bottom left**) and emission (**bottom right**) maxima for separate solutions containing squaraine or macrocycle complex in water.

Preliminary threading studies in water were performed by mixing separate solutions of water-soluble squaraines **S1PEG**, **S2PEG** and **S3PEG** with one molar equivalent of water-soluble macrocycle **M2**. Shown in Figure 4 is a photograph of the different samples and also the absorption and emission maxima. The absorption spectra for free **S1PEG**, and especially **S2PEG** and **S3PEG**, exhibit band broadening due to self-aggregation of the dyes. But as hoped, macrocycle threading produced three changes in spectral properties: (a) A significant sharpening of the squaraine absorption bands, (b) a 20–35 nm red-shift in the squaraine absorption and emission maxima wavelength, and (c) a significant increase in fluorescence quantum yield.

**Figure 5:**
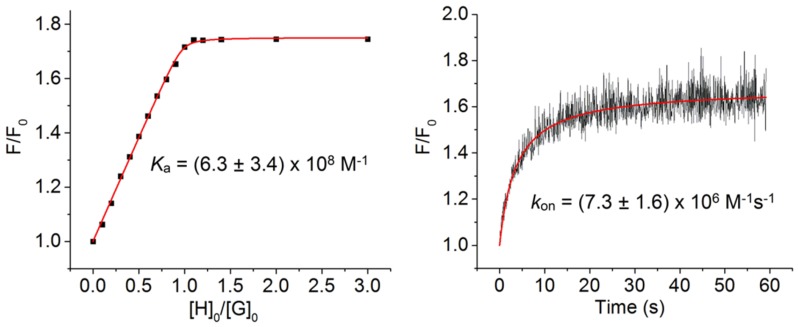
(**left**) Fluorescence (ex: 809 nm, em: 829 nm, slit 5 nm) titration isotherm for incremental addition of **M2** to a solution of **S3PEG** (250 nM) in H_2_O at 25 °C. (**right**) Threading kinetic profile generated by mixing equal molar concentration (50 nM each) of **S3PEG** and **M2** in a stopped flow device (ex: 809 nm, em: 829 nm, slit 5 nm). The red lines are computer fits of experimental data to 1:1 binding model or second order kinetic model, respectively.

The association constants for threading of **M2** by **S1PEG**, **S2PEG** or **S3PEG** in water were measured by fluorescence titration (Figure 5, Figures S1 and S2) and the values of K_a_ are listed in Table 1. The symmetrical thienothiophene-based squaraine **S3PEG** has essentially the same nanomolar affinity for **M2** as analogous but shorter symmetrical thiophene-based squaraines [14]. Although the extended chromophore in **S3PEG** has a larger hydrophobic surface area, it does not translate into higher affinity for **M2**, presumably because there is no increase in the amount of hydrophobic surface area that is buried by complexation (see the molecular model in Figure 3). The K_a_ values for unsymmetrical **S1PEG** and **S2PEG** are 7.8 and 5.3 times higher than **S3PEG** because these dyes have terminal *N*,*N*-dibenzyl groups which are known to stabilize the threaded complexes by stacking with peripheral surfaces of the surrounding macrocycle [29]. For each water-soluble squaraine, the second order rate constant for macrocycle threading, k_on_, was measured using a stopped flow device that monitored the increase in fluorescence after mixing the squaraine and macrocycle. As shown in Figure 5, threading of **M2** by **S3PEG** was complete in less than a minute when the concentration of each binding partner was 250 nM. Compared to **S3PEG**, the threading of **M2** by **S1PEG** and **S2PEG** (Figures S1 and S2) was 25 and 100 times faster, respectively, which was expected based on the known dependence of k_on_ on the steric size of *N*-alkyl groups at the end of the squaraine chromophore [17]. Squaraines **S1PEG** and **S2PEG** have *N*-methyl groups whereas squaraine **S3PEG** has larger *N*-ethyl groups which were needed to increase the dye solubility. 

### 2.4. Photophysical Properties

In Table 2 is a summary of the photophysical properties of the six different dye systems in water. The photostability of each squaraine and its macrocycle complex in water was monitored by continuous NIR light excitation over 15 h and in each case there was no or little loss in sample fluorescence intensity (Figures S3–S5). The chemical stabilities of the dyes and their macrocycle complexes were tested by monitoring the fluorescence intensity over time in the presence of strongly nucleophilic Na_2_S in water (Figures S6–S8). All three dyes were quickly bleached by the Na_2_S, whereas the corresponding macrocycle complexes were quite stable, which is compelling evidence that the encapsulated squaraines were inside the protective macrocycle **M2**. This combination of high resistance to chemical and photobleaching exhibited by the complexes is quite notable for such highly π-extended chromophores [3].

**Figure 6:**
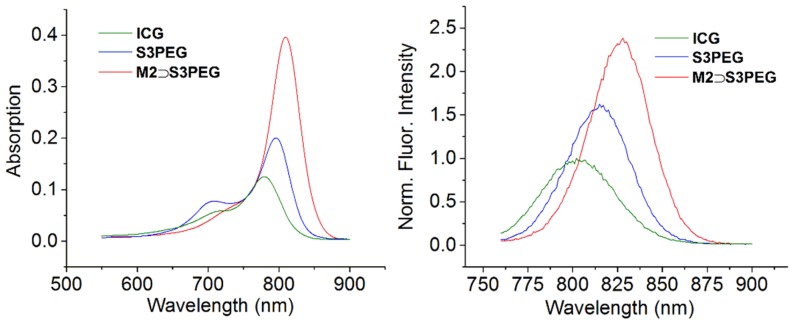
(**left**) Absorbance and (**right**) emission spectra (ex: 750 nm) of **ICG**, **S3PEG**, **M2** ⊃ **S3PEG** with concentration adjusted to give identical absorbance values (0.08) at 750 nm in H_2_O.

Presently, the only NIR dye that is approved for use in humans is indocyanine green (**ICG**). Although **ICG** is employed extensively, it is also known to have nonoptimal performance properties and there is an active community effort to find replacement dyes with very similar absorption/emission wavelengths. Shown in Figure 6 is a comparison of the absorption and emission maxima for **ICG**, **S3PEG** and **M2** ⊃ **S3PEG** in water. The absorption spectra for **ICG** and **S3PEG** both exhibited blue-shifted aggregation bands whereas the maxima band for **M2** ⊃ **S3PEG** was relatively sharp and narrow (Figure 6). To compare the fluorescence quantum yields, the concentrations of each sample were adjusted to produce the same absorbance value (0.08) at 750 nm, and the fluorescent emission spectrum was collected with excitation at 750 nm. Integration of the emission bands in Figure 6 indicated that the fluorescence quantum yields for **S3PEG** and **M2** ⊃ **S3PEG** were 1.3 and 2 times higher than **ICG** (Table S1).

**Figure 7:**
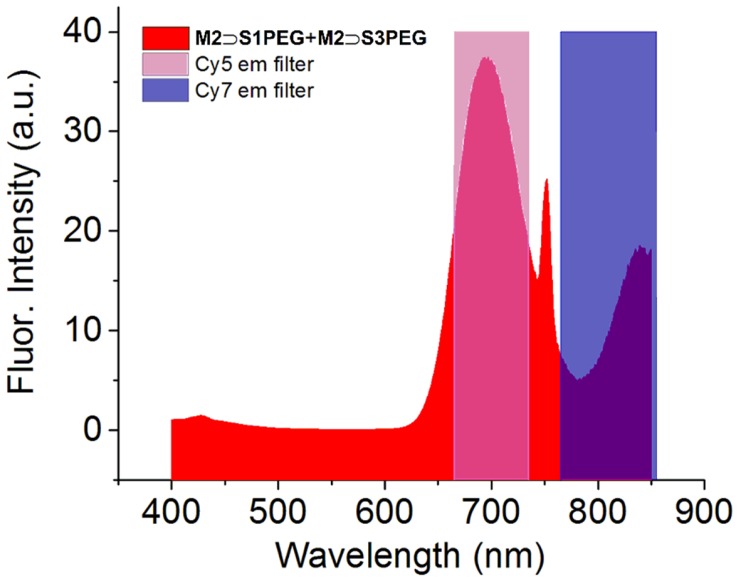
Two color emission by single excitation (375 nm) of a 1:1 mixture containing **M2** ⊃ **S1PEG** and **M2** ⊃ **S3PEG** in H_2_O. The peak at 750 nm is an artifact caused by the excitation at 375 nm.

A unique photophysical feature of these threaded squaraine macrocycle complexes is the option of squaraine excitation by internal energy transfer [14]. That is, 375 nm excitation of the anthracene sidewalls of the surrounding macrocycle (**M2**) is followed by efficient internal energy transfer within the complex and excitation of the encapsulated squaraine. This capability raises the possibility of multiplex excitation of multiple dyes in the same sample. Shown in Figure 7 is the two-color emission spectrum that was observed when 1:1 mixture of **M2** ⊃ **S1PEG** and **M2** ⊃ **S3PEG** in water was excited at 375 nm. The two emission bands nicely match the popular Cy5 and Cy7 emission channels that are commonly used for biological imaging. Single wavelength excitation and multiplex detection is a valuable tool in orthogonal fluorescence imaging, and is likely to be very helpful in technologies such as fluorescence-guided surgery and cancer tissue histology, where there is a desire to simultaneously identify the location of different molecular probes that label distinct biological targets [31,32].

## 3. Materials and Methods

### 3.1. General

^1^H and ^13^C-NMR spectra were recorded on Bruker AVANCE III HD 400 (Billerica, MA, USA) and 500 MHz spectrometer (Billerica, MA, USA) Chemical shift was presented in ppm and referenced by residual solvent peak. Mass spectrometry (MS) was either performed using a Bruker microTOF II spectrometer (Billerica, MA, USA) with electron spray ionization (ESI) or Bruker Autoflex III (Billerica, MA, USA) with matrix-assisted laser desorption/ionization (MALDI). Commercially available solvents and chemicals were used without further purification unless otherwise stated. Water was de-ionized and micro filtered. Flash column chromatography was performed using Biotage flash column chromatography purification system with SNAP Ultra cartridges (Charlotte, NC, USA). All cartridges used silica gel as stationary phase unless otherwise stated.

### 3.2. Synthesis 

Compound **1** [17], **2** [33] and **3** [34] were prepared using previously reported literature methods.

**S1**: Compound **1** (80 mg, 0.410 mmol) and compound **2** (150 mg, 0.406 mmol) were dissolved in a mixture of 1-butanol (15 mL) and benzene (45 mL) and the reaction mixture was heated to reflux for 2 h with Dean-Stark distillation. The solvent was removed and the residue was purified by column chromatography using 0–10% MeOH/CHCl_3_ to produce pure **S1** as a blue solid (172 mg, 77% yield). ^1^H-NMR (500 MHz, CDCl_3_) δ 8.15–8.12 (m, 2H), 8.11 (s, 1H), 7.32 (dd, *J* = 8.1, 6.6 Hz, 4H), 7.29–7.24 (m, 2H), 7.23–7.17 (m, 4H), 6.79 (d, *J* = 9.1 Hz, 2H), 6.47 (d, *J* = 5.1 Hz, 1H), 4.73 (s, 4H), 4.11 (d, *J* = 2.4 Hz, 2H), 3.77 (s, 2H), 3.70 (t, *J* = 4.9 Hz, 2H), 3.26 (s, 3H), 2.41 (t, *J* = 2.4 Hz, 1H). ^13^C-NMR (126 MHz, CDCl_3_) δ 181.00, 180.74, 179.96, 176.30, 173.77, 152.09, 142.94, 137.23, 130.80, 129.10, 128.89, 127.60, 126.72, 121.30, 115.67, 113.55, 112.79, 79.08, 75.59, 66.79, 58.79, 56.34, 54.25, 43.10. HRMS-ESI *m*/*z* 547.2026 ([M + H]^+^,C_34_H_31_N_2_O_3_S^+^, calc. 547.2050).

**S1PEG**: Compound **S1** (12 mg, 0.021 mmol), azido-mPEG_45_ **3** (40 mg, 0.020 mmol), triethylamine (2 drops) and TBTACu(I)Br (2 mg) were dissolved in CHCl_3_ (5 mL) and the reaction mixture was stirred at room temperature for 12 h. After removing the solvent, the residue was purified by column chromatography using 0–10% MeOH/CHCl_3_ (containing 0.2% NH_4_OH) to obtain pure **S1PEG** as a blue solid (48 mg, 92% yield). ^1^H-NMR (500 MHz, CDCl_3_) δ 8.18 (d, *J* = 5.0 Hz, 1H), 8.15 (d, *J* = 9.2 Hz, 2H), 7.70 (s, 1H), 7.37–7.31 (m, 4H), 7.30–7.27 (m, 2H), 7.23–7.18 (m, 4H), 6.81 (d, *J* = 9.2 Hz, 2H), 6.50 (d, *J* = 5.0 Hz, 1H), 4.73 (s, 4H), 4.62 (s, 2H), 4.52 (dd, *J* = 5.5, 4.6 Hz, 2H), 3.87–3.81 (m, 4H), 3.79–3.72 (m, 2H), 3.68–3.46 (m, 203H), 3.37 (s, 3H), 3.30 (s, 3H). MS-MALDI (DHBA as matrix) showed a set of peaks around 2363 which reflected the polydispersity of the PEG_45_ chains.

*2-Bromothieno[3,2-b]thiophene* (**4**): Thieno[3,2-*b*]thiophene (1.0 g, 7.13 mmol) was dissolved in acetic acid (10 mL). *N*-bromosuccinimide (1.27 g, 7.13 mmol) was added and the reaction mixture was stirred at room temperature for 2 h. Solvent was removed and the residue was dissolved in diethyl ether (80 mL) and washed with NaOH (1 M, 100 mL × 3), H_2_O (100 mL × 3) and brine (100 mL). The resulting solution was dried over Na_2_SO_4_, and the solvent was removed by rotary evaporation to obtain pure **4** as a light yellow liquid (1.3 g, 81% yield). ^1^H-NMR (500 MHz, CDCl_3_) δ 7.40 (ddd, *J* = 5.7, 3.0, 1.5 Hz, 1H), 7.28 (d, *J* = 4.1 Hz, 1H), 7.20–7.14 (m, 1H). ^13^C-NMR (126 MHz, CDCl_3_) δ 127.57, 126.78, 122.32, 121.98, 119.60, 119.24. HRMS-ESI *m*/*z* 217.8837 ([M]^+^, C_6_H_3_BrS_2_^+^, calc. 217.8860).

*2-(methyl(thieno[3,2-b]thiophen-2-yl)amino)ethan-1-ol* (**5**): Compound **4** (1.3 g, 5.8 mmol), Cu^0^ (70 mg, 1.2 mmol), CuI (220 mg, 1.2 mmol) and K_3_PO_4_·H_2_O (2.7 g, 11.6 mmol) were suspended in 2-(methylamino)ethanol (15 mL). The reaction mixture was stirred at 90 °C over 5 h. After cooling to room temperature, H_2_O (80 mL) was added, and the reaction mixture was extracted with diethyl ether (100 mL × 3). The organic phase was combined and dried over Na_2_SO_4_, the solvent was removed and the residue was purified by column chromatography (neutral Al_2_O_3_ as stationary phase) using 10–40% EtOAc/hexane to obtain pure **5** as a light yellow solid (570 mg, 46% yield). ^1^H-NMR (500 MHz, Acetone-*d*_6_) δ 7.10 (dd, *J* = 5.2, 0.6 Hz, 1H), 7.07 (d, *J* = 5.2 Hz, 1H), 6.12 (d, *J* = 0.7 Hz, 1H), 3.99 (t, *J* = 5.5 Hz, 1H), 3.80 (q, *J* = 5.7 Hz, 2H), 3.40 (t, *J* = 5.9 Hz, 2H), 3.00 (s, 3H). ^13^C-NMR (126 MHz, Acetone-*d*_6_) δ 206.11, 160.53, 139.67, 120.16, 119.69, 93.96, 59.29, 57.85, 40.43, 29.67, 29.51, 29.36, 29.21, 29.05. HRMS-ESI *m*/*z* 214.0379 ([M + H]^+^, C_9_H_12_NOS_2_^+^, calc. 214.0355).

*N-methyl-N-(2-(prop-2-yn-1-yloxy)ethyl)thieno[3,2-b]thiophen-2-amine* (**6**): Propargyl bromide (900 µL, 8.0 mmol) was added to a mixture of compound **5** (570 mg, 2.7 mmol), aqueous NaOH (15 mL, 50% w%) and toluene (15 mL). TBA HSO_4_ (200 mg) was added to the mixture as a phase transfer reagent and the resulting solution was stirred at room temperature for 5 h. Toluene was removed by rotary evaporation and H_2_O (100 mL) was added to the residue. The resulting solution was extracted with diethyl ether (100 mL × 3). The organic phase was combined and dried over Na_2_SO_4_, the solvent was removed and the residue was purified by column chromatography (neutral Al_2_O_3_ as stationary phase) using 5–20% EtOAc/hexane to obtain pure **6** as a yellow liquid (650 mg, 97% yield). ^1^H-NMR (500 MHz, Acetone-*d*_6_) δ 7.11 (dd, *J* = 5.2, 0.6 Hz, 1H), 7.09 (d, *J* = 5.2 Hz, 1H), 6.16 (d, *J* = 0.6 Hz, 1H), 4.18 (d, *J* = 2.4 Hz, 4H), 3.75 (t, *J* = 5.6 Hz, 4H), 3.49 (t, *J* = 5.6 Hz, 4H), 3.00 (s, 6H), 2.93 (t, *J* = 2.4 Hz, 2H). ^13^C-NMR (126 MHz, Acetone-*d*_6_) δ 205.53, 160.12, 139.58, 125.88, 120.28, 119.63, 94.26, 80.10, 75.36, 67.20, 58.03, 54.88, 40.36. HRMS-ESI *m*/*z* 252.0540 ([M + H]^+^, C_12_H_14_NOS_2_^+^, calc. 252.0511).

**S2**: Compound **6** (148 mg, 0.4 mmol) and 3-(4-(dibenzylamino)phenyl)-4-hydroxycyclobut-3-ene-1,2-dione (100 mg, 0.4 mmol) were dissolved in a mixture of 1-butanol (15 mL) and benzene (45 mL). The reaction was heated to reflux with Dean-Stark distillation for 2 h. After cooling to room temperature, the solvent was removed and the residue was purified by column chromatography using 0–7% MeOH/CHCl_3_ to obtain pure **S2** as green solid (140 mg, 58% yield). ^1^H-NMR (500 MHz, CDCl_3_) δ 8.28–8.14 (m, 3H), 7.39–7.27 (m, 4H), 7.25–7.13 (m, 6H), 6.85 (d, *J* = 8.9 Hz, 2H), 6.23 (s, 1H), 4.77 (s, 4H), 4.17 (d, *J* = 2.4 Hz, 2H), 3.81 (t, *J* = 4.8 Hz, 2H), 3.67 (d, *J* = 6.4 Hz, 2H), 3.25 (s, 3H), 2.44 (t, *J* = 2.3 Hz, 1H). ^13^C-NMR: Limited compound solubility prevented ^13^C analysis. HRMS-ESI *m*/*z* 602.1704 ([M]^+^, C_36_H_30_N_2_O_3_S_2_^+^, calc. 602.1698).

**S2PEG**: Compound **S2** (16 mg, 0.026 mmol), azido-mPEG_45_ **3** (50 mg, 0.024 mmol), triethylamine (2 drops) and TBTACu(I)Br (2 mg) was dissolved in CHCl_3_ (5 mL) and the reaction mixture was stirred at room temperature for 12 h. After removing the solvent, the residue was purified by column chromatography (silica gel as stationary phase) using 0–10% MeOH/CHCl_3_ to obtain the crude product. The crude product was further purified by a second column (neutral Al_2_O_3_ as stationary phase) using 0–5% MeOH/CHCl_3_ to obtain pure **S2PEG** as green solid (35 mg, 55% yield). ^1^H-NMR (500 MHz, CDCl_3_) δ 8.23–8.20 (m, 3H), 7.69 (s, 1H), 7.35 (dd, *J* = 8.1, 6.6 Hz, 4H), 7.29 (d, *J* = 7.3 Hz, 2H), 7.23–7.18 (m, 5H), 6.84 (d, *J* = 9.2 Hz, 2H), 6.23 (s, 1H), 4.76 (s, 5H), 4.64 (s, 2H), 4.54–4.46 (m, 3H), 3.84–3.71 (m, 6H), 3.70–3.48 (m, 196H), 3.37 (s, 3H), 3.20 (s, 3H). MS-MALDI (DHBA as matrix) showed a set of peaks around 2421, which reflected the polydispersity of the PEG_45_ chains.

*2-(ethyl(thieno[3,2-b]thiophen-2-yl)amino)ethan-1-ol* (**7**): Compound **4** (1.3 g, 5.8 mmol), Cu^0^ (70 mg, 1.2 mmol), CuI (220 mg, 1.2 mmol) and K_3_PO_4_·H_2_O (2.7 g, 11.6 mmol) was suspended in 2-(ethylamino)ethanol (15 mL). The reaction mixture was stirred at 90 °C over 5 h. After cooling to room temperature, H_2_O (80 mL) was added, and the reaction mixture was extracted with diethyl ether (100 mL × 3). The organic phase was combined and dried over Na_2_SO_4_, the solvent was removed and the residue was purified by column chromatography (neutral Al_2_O_3_ as stationary phase) using 10–40% EtOAc/hexane to obtain pure **7** as light yellow solid (660 mg, 51% yield). ^1^H-NMR (500 MHz, Acetone-*d*_6_) δ 7.10 (dd, *J* = 5.2, 0.6 Hz, 1H), 7.07 (d, *J* = 5.2 Hz, 1H), 6.17 (d, *J* = 0.7 Hz, 1H), 3.97–3.81 (m, 1H), 3.76 (t, *J* = 6.1 Hz, 2H), 3.45–3.35 (m, 4H), 1.19 (t, *J* = 7.1 Hz, 3H). ^13^C-NMR (126 MHz, Acetone-*d*_6_) δ 205.64, 159.34, 139.55, 120.06, 119.63, 94.31, 59.32, 55.29, 48.45, 29.53, 29.38, 29.22, 29.07, 28.92, 11.67. HRMS-ESI m/z 228.0534 ( [M + H]^+^, C_10_H_14_NOS_2_^+^, calc. 228.0511).

*N-ethyl-N-(2-(prop-2-yn-1-yloxy)ethyl)thieno[3,2-b]thiophen-2-amine* (**8**): Propargyl bromide (900 µL, 8.0 mmol) was added to a mixture of compound **7** (660 mg, 2.9 mmol) in toluene (15 mL) and NaOH solution (15 mL, 50% w%). TBA HSO_4_ (200 mg) as phase transfer reagent was added to the mixture and the resulting solution was stirred at room temperature for 5 h. Toluene was removed by rotary evaporation and H_2_O (100 mL) was added to the residue. The resulting solution was extracted with diethyl ether (100 mL × 3). Organic phase was combined and dried over Na_2_SO_4_, the solvent was removed and the residue was purified by column chromatography (neutral Al_2_O_3_ as stationary phase) using 5–20% EtOAc/hexane to obtain pure **8** as a yellow liquid (520 mg, 84% yield). ^1^H-NMR (500 MHz, Acetone-*d*_6_) δ 7.11 (dd, *J* = 5.2, 0.6 Hz, 1H), 7.08 (d, *J* = 5.1 Hz, 1H), 6.18 (d, *J* = 0.5 Hz, 1H), 4.18 (d, *J* = 2.4 Hz, 4H), 3.72 (t, *J* = 5.8 Hz, 4H), 3.48 (t, *J* = 5.8 Hz, 4H), 3.39 (q, *J* = 7.1 Hz, 4H), 2.93 (t, *J* = 2.4 Hz, 2H), 1.19 (t, *J* = 7.1 Hz, 6H). ^13^C-NMR (126 MHz, Acetone-*d*_6_) δ 205.55, 159.01, 139.54, 125.67, 120.24, 119.65, 94.55, 80.14, 75.39, 67.45, 58.09, 52.51, 48.43, 29.42, 29.27, 29.12, 11.82. HRMS-ESI *m*/*z* 266.0685 ([M + H]^+^, C_13_H_16_NOS_2_^+^, calc. 266.0688).

**S3**: Compound **8** (150 mg, 0.57 mmol), 3,4-dihydroxycyclobut-3-ene-1,2-dione (32 mg, 0.28 mmol) was dissolved in a mixture of 1-butanol (15 mL) and benzene (45 mL). The reaction was heated to reflux with Dean-Stark distillation for 2 h. After cooling to room temperature, the solvent was removed and the residue was purified by recrystallization in MeOH to obtain pure **S3** as a brown solid (158 mg, 92% yield). ^1^H-NMR (500 MHz, CDCl_3_) δ 8.11 (s, 2H), 6.19 (s, 2H), 4.18 (d, *J* = 2.4 Hz, 4H), 3.81 (d, *J* = 5.2 Hz, 4H), 3.67 (d, *J* = 5.4 Hz, 4H), 3.22 (s, 6H), 2.45 (t, *J* = 2.4 Hz, 2H). ^13^C-NMR: Limited compound solubility prevented ^13^C analysis. HRMS-ESI m/z 608.0921 ([M]^+^, C_30_H_28_N_2_O_4_S_4_^+^, calc. 608.0932).

**S3PEG**: Compound **S3** (7.0 mg, 0.012 mmol), azido-mPEG_45_ **3** (50 mg, 0.024 mmol), triethylamine (2 drops) and TBTACu(I)Br (2 mg) were mixed in CHCl_3_ (5 mL) and the reaction mixture was sonicated at 40 °C for 4 h. After removing the solvent, the residue was purified by column chromatography (silica gel as stationary phase) using 0–10% MeOH/CHCl_3_ to obtain the crude product. The crude product was further purified by a send column chromatography (neutral Al_2_O_3_ as stationary phase) using 0–5% MeOH/CHCl_3_ to obtain pure **S3PEG** as a brown solid (24 mg, 42% yield). ^1^H-NMR (500 MHz, CDCl_3_) δ 8.00 (s, 2H), 7.67 (s, 2H), 6.13 (s, 2H), 4.60 (s, 4H), 4.47 (dd, *J* = 5.5, 4.5 Hz, 4H), 3.79 (dd, *J* = 5.5, 4.5 Hz, 4H), 3.74 (d, *J* = 5.4 Hz, 4H), 3.66–3.39 (m, 429H), 3.33 (s, 6H), 1.22 (t, *J* = 7.2 Hz, 6H). MS-MALDI (DHBA as matrix) showed a set of peaks around 4391 which reflected the polydispersity of the PEG_45_ chains.

### 3.3. Association and Kinetic Measurements

*Association Measurements*: Stock solutions of squaraine guest, generally 0.03–3 µΜ were prepared and stock solutions of the host (0.6–60 µM) were made by using the guest solution as the solvent (to keep the concentration of guest constant during the titration). A solution of guest (1 mL) was placed in the cuvette and host solution was titrated into guest solution. Spectra changes were recorded by fluorometer after each injection. Association constants were determined using Origin Lab^TM^ 8.6 software (Northampton, MA, USA) that enabled non-linear least squares fitting of the titration data with an equation for 1:1 binding.

*Kinetic Measurements:* Kinetic studies were performed by using a SFA-20M stopped flow device (empirical dead time < 8 ms) (Kyoto, Japan). Equal volumes of host solution and guest solution were mixed by the stopped flow device. The spectral changes were monitored by fluorescence. Second order rates constants were determined using Origin Lab^TM^ 8.6 software that enabled non-linear least squares fitting of the titration data with an equation for second order kinetics.

## 4. Conclusion

A new family of fluorescent thiophene and thienothiophene-containing squaraine dyes was prepared using simple synthesis and purification methods. Each squaraine can be rapidly encapsulated by a tetralactam macrocycle in organic or aqueous solution. The threaded supramolecular complexes have nanomolar affinity in water, increased stability towards chemical attack by nucleophiles, and favorable optical properties such as increased fluorescence quantum yield and decreased fluorescence quenching upon dye self-aggregation. Together, the set of squaraine dyes and their macrocycle complexes cover the absorption/emission range of 600–800 nm. A notable favorable attribute with the threaded complexes is the capability to excite each of them with 375 nm light and produce an emission band that is characteristic of the encapsulated squaraine. A wide set of single wavelength and multiplex detection techniques are envisioned using these new dyes for various practical applications in diagnostics, microscopy, molecular imaging, and fluorescence-guided surgery.

## Figures and Tables

**Table 1:** Association constant (*K*_a_) and rate constant (*k*_on_) for squaraine threading of **M2** in H_2_O at 25 °C.

	*K*_a_ (M^−1^)	*k*_on_ (M^−1^s^−1^)
**S1PEG**	(4.9 ± 2.0) × 10^9^	(1.9 ± 0.2) × 10^8^
**S2PEG**	(3.4 ± 1.5) × 10^9^	(7.0 ± 1.0) × 10^8^
**S3PEG**	(6.3 ± 3.4) × 10^8^	(7.3 ± 1.6) × 10^6^

**Table 2:** Photophysical properties of squaraines and their complexes with **M2** in H_2_O.

	S1PEG	M2 ⊃ S1PEG	S2PEG	M2 ⊃ S2PEG	S3PEG	M2 ⊃ S3PEG
Abs (nm)	608	638	695	722	795	809
Em (nm)	665	697	731	753	815	829
Φ*_f_^a^*	6%	25%	3%	19%	7%	11%

Quantum yields of **S1PEG** and **M2** ⊃ **S1PEG** were measured relative to bis(*N*,*N*-dimethylaminophenyl)squaraine, quantum yields of **S2PEG** and **M2** ⊃ **S2PEG** were measured relative to cryptocyanine, and quantum yields of **S3PEG** and **M2** ⊃ **S3PEG** were measured relative to **ICG** [30].

## Supplementary Materials

The following are available online, Figure S1: (left) Fluorescence (ex: 630 nm, em: 700 nm, slit 3 nm) titration isotherm for incremental addition of **M2** to a solution of **S1PEG** (250 nM) in water. (right) Threading kinetic profile generated by mixing equal molar concentration (50 nM each) of **S1PEG** and **M2** in a stopped flow device (ex: 630 nm, em: 700 nm, slit 3 nm), Figure S2: (left) Fluorescence (ex: 723 nm, em: 753 nm, slit 5 nm) titration isotherm for incremental addition of **M2** to a solution of **S2PEG** (250 nM) in water. (right) Threading kinetic profile generated by mixing equal molar concentration (50 nM each) of **S2PEG** and **M2** in a stopped flow device (ex: 723 nm, em: 753 nm, slit 3 nm), Figure S3: Photostability of free **S1PEG** (5 µM, left) and **M2** ⊃ **S1PEG** (5 µM, right) in H_2_O with continuous irradiation at 550 nm over 15 h (fluorescence spectrum was collected every 30 min), Figure S4. Photostability test of free **S2PEG** (2 µM, left) and **M2** ⊃ **S3PEG** (2 µM, right) in H_2_O with continuous irradiation at 650 nm over 15 h (fluorescence spectrum was collected every 30 min), Figure S5. Photo stability test of the **S3PEG** (2 µM, left) and **M2** ⊃ **S3PEG** (2 µM, right) in H_2_O with continuous irradiation at 750 nm over 15 h (fluorescence spectrum was collected every 30 min), Figure S6. Chemical stability test of **S1PEG** and **M2** ⊃ **S1PEG**. (left) Change in fluorescent maxima band for solutions of (A) **M2** ⊃ **S1PEG** (5.0 µM) or (B) free **S1PEG** (5 µM), in the presence of excess nucleophile Na_2_S (5 mM) in water at 20°C. (right) Photograph of samples containing, (A) **M2** ⊃ **S1PEG** (80 µM) or (B) free **S1PEG** (80 µM), after sitting in the presence of excess nucleophile Na_2_S (100 mM) in water at 20 °C, Figure S7. Chemical stability test of **S2PEG** and **M2** ⊃ **S2PEG** by monitoring the change in fluorescent maxima band for solutions of (A) **M2** ⊃ **S2PEG** (5.0 µM ) or (B) free **S2PEG** (5 µM) over time in the presence of excess nucleophile Na_2_S (5 mM) in water at 20 °C, Figure S8. Chemical stability test of **S3PEG** and **M2** ⊃ **S3PEG** by monitoring change in fluorescent maxima band for solutions of (A) **M2** ⊃ **S3PEG** (1.0 µM) or (B) free **S3PEG** (1 µM), in the presence of excess nucleophile Na_2_S (1 mM) in water at 20 °C, Table S1. Integrated fluorescent and quantum yield of **ICG**, **S3PEG**, and **M2** ⊃ **S3PEG** in H_2_O. Table S2. Photophysical properties of squaraines and their complexes with **M1** in CHCl_3_.
