# Supplementary material for: Fluorescent Thienothiophene-Containing Squaraine Dyes and Threaded Supramolecular Complexes with Tunable Wavelengths between 600–800 nm
Source: Molecules. 2018 Sep 1;23(9):2229. doi: 10.3390/molecules23092229 (PMC6225225; doi:10.3390/molecules23092229)
Supplement: Supplementary file 1 [file molecules-23-02229-s001.pdf]

Supporting Information for

Fluorescent Thienothiophene-Containing Squaraine Dyes and Threaded Supramolecular Complexes with Tunable Wavelengths between 600-800 nm

Wenqi Liu, Hannah H. McGarraugh, and Bradley D. Smith*
Department of Chemistry and Biochemistry,
236 Nieuwland Science Hall,
University of Notre Dame, Notre Dame, IN 46556, USA
*smith.115@nd.edu

A. Compound Characterization
B. Titration Data
C. Photophysical Data
D. Chemical Stability Data
E. Fluorescence Quantum Yields

A. Compound Characterization

The peaks assignments below are based on precedent and logic. They have not been confirmed by correlation methods.

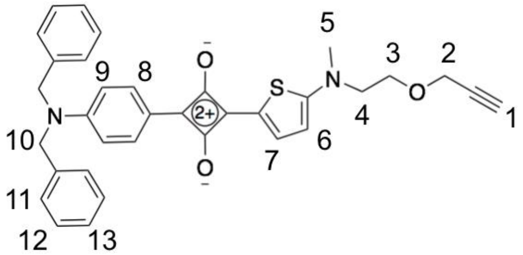

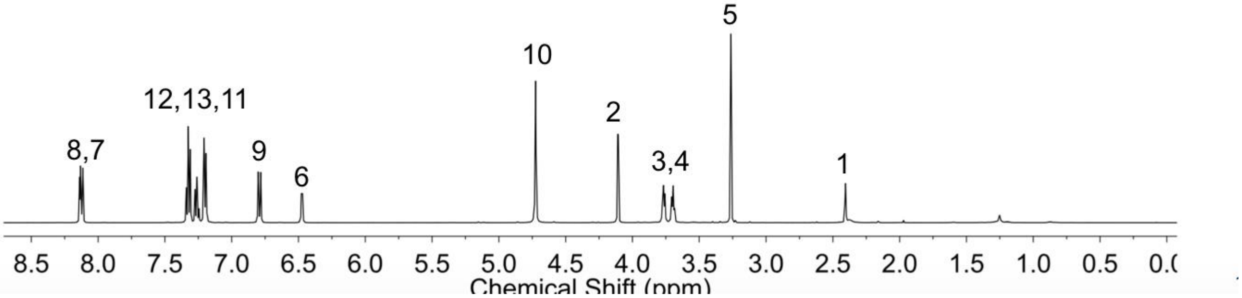

^{1}H NMR (500 MHz, $CDCl_3$) of **S1**.

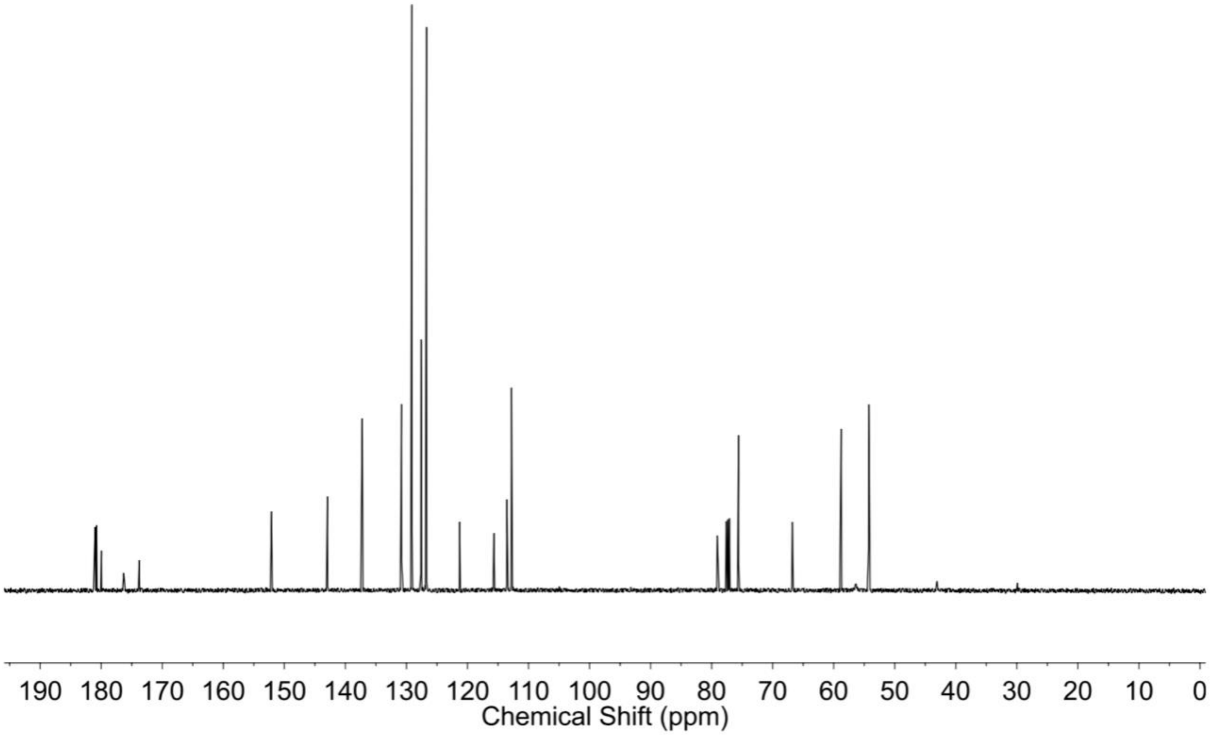

^{13}C NMR (126 MHz, $CDCl_3$) of **S1**.

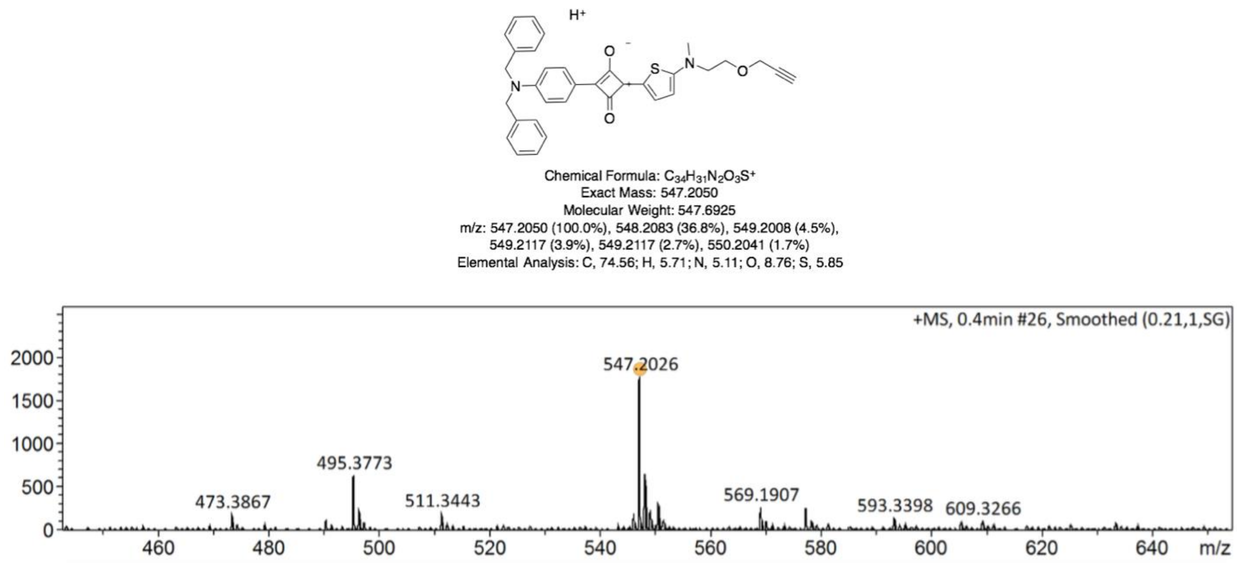

HRMS-ESI of **S1**.

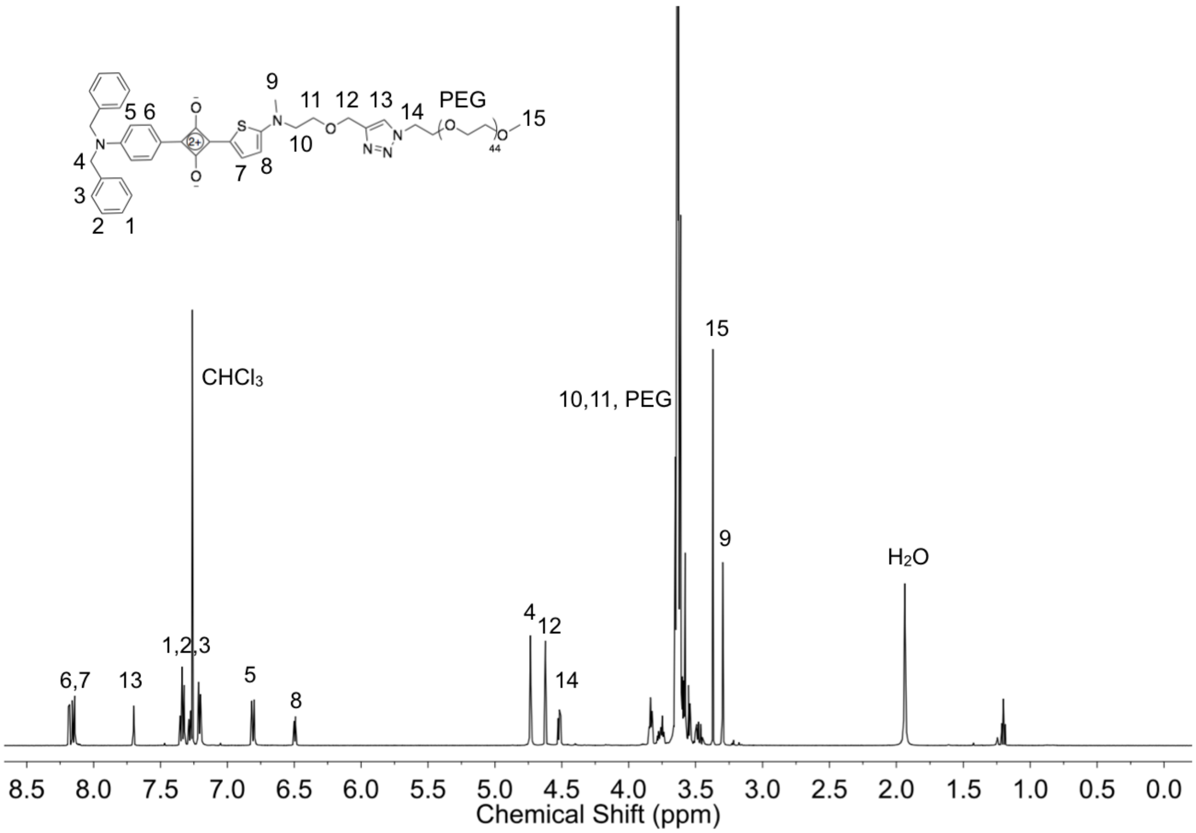

^{1}H NMR (500 MHz, $CDCl_3$) of **S1PEG**.

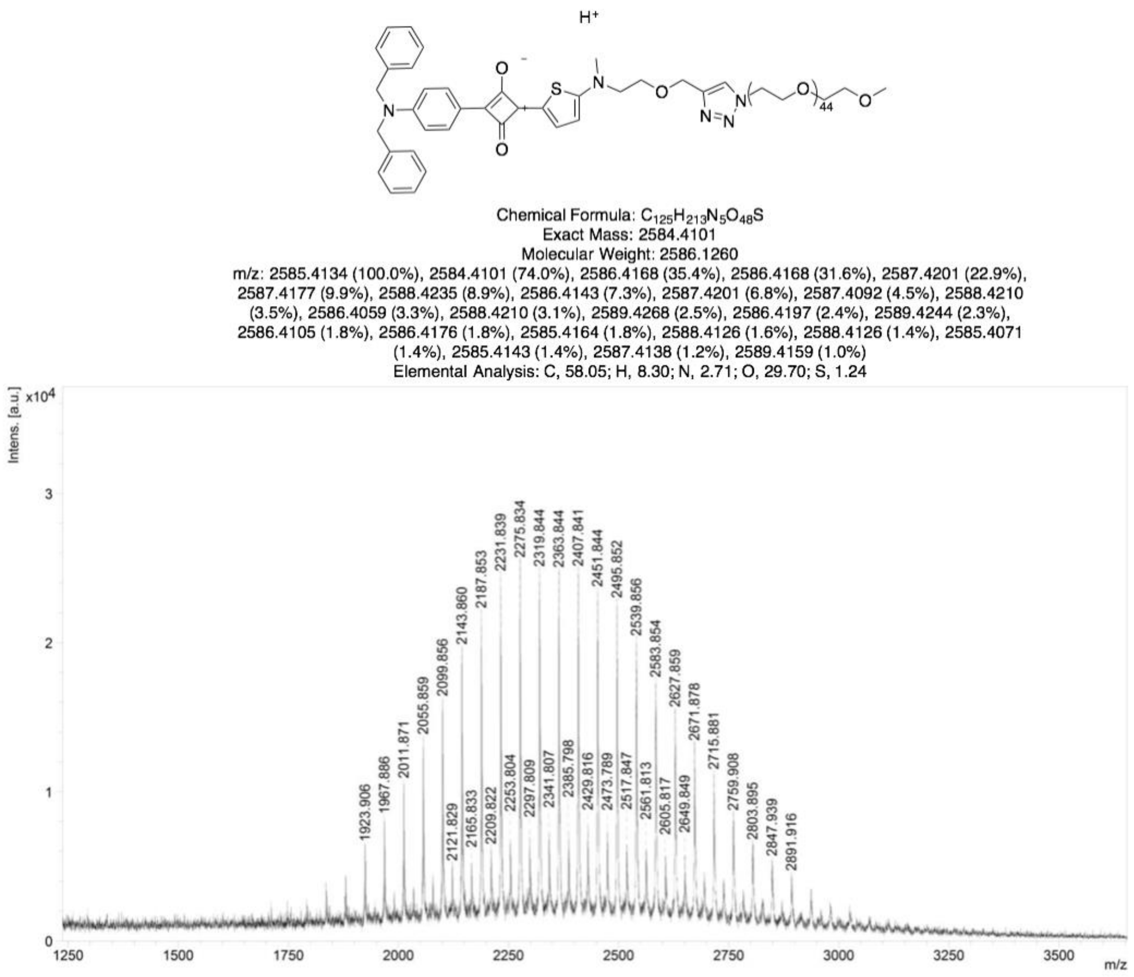

MS-MALDI (DHBA as matrix) of **S1PEG**. A set of peaks around 2363 reflect the polydispersity of the PEG_{45} chains.

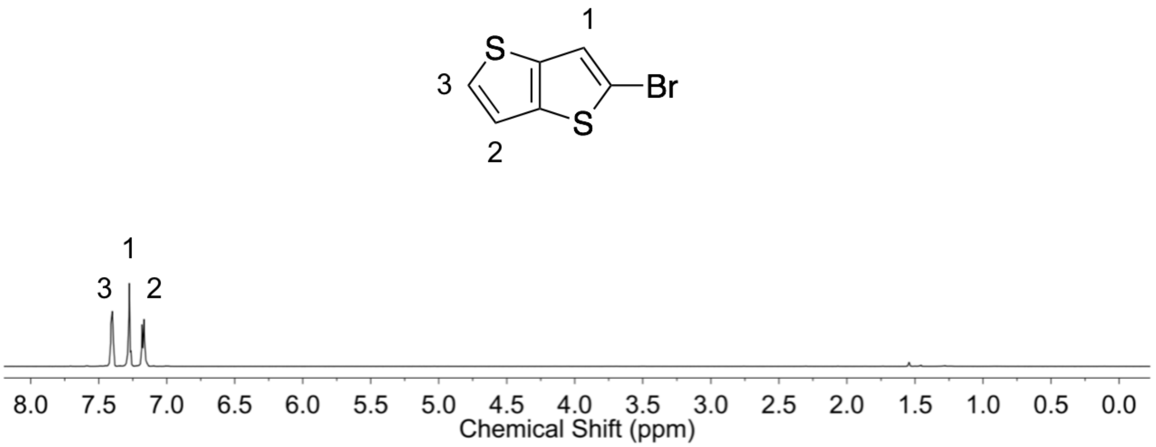

^{1}H NMR (500 MHz, $CDCl_3$) of **4**.

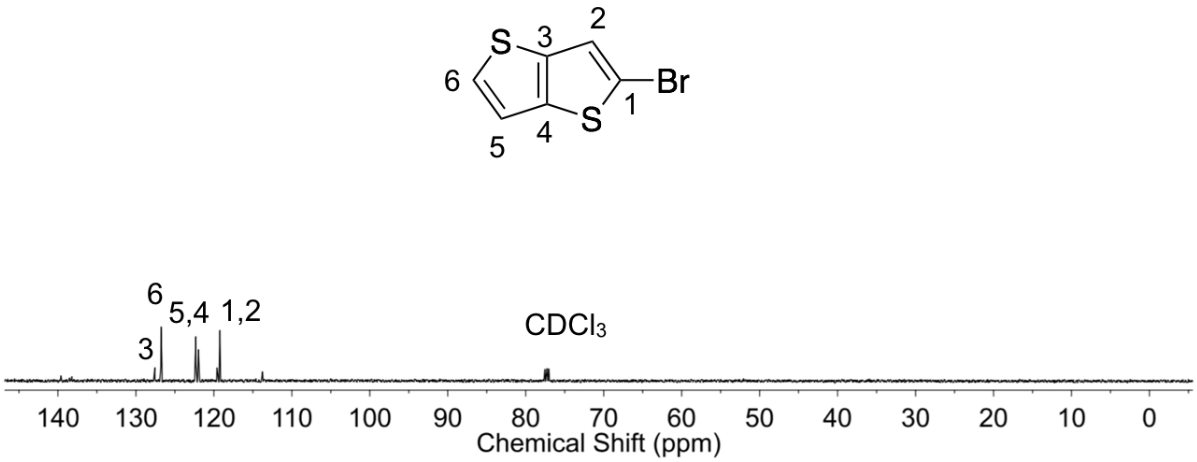

^{13}C NMR (126 MHz, $CDCl_3$) of **4**.

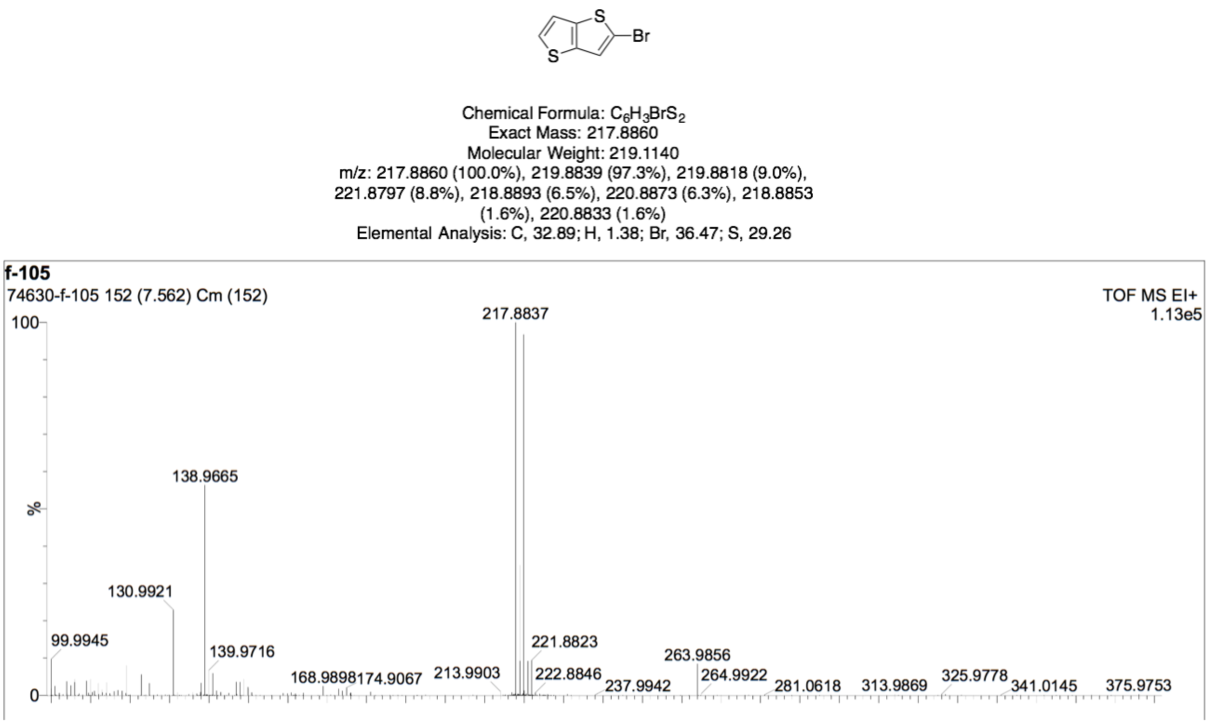

HRMS-ESI of **4**.

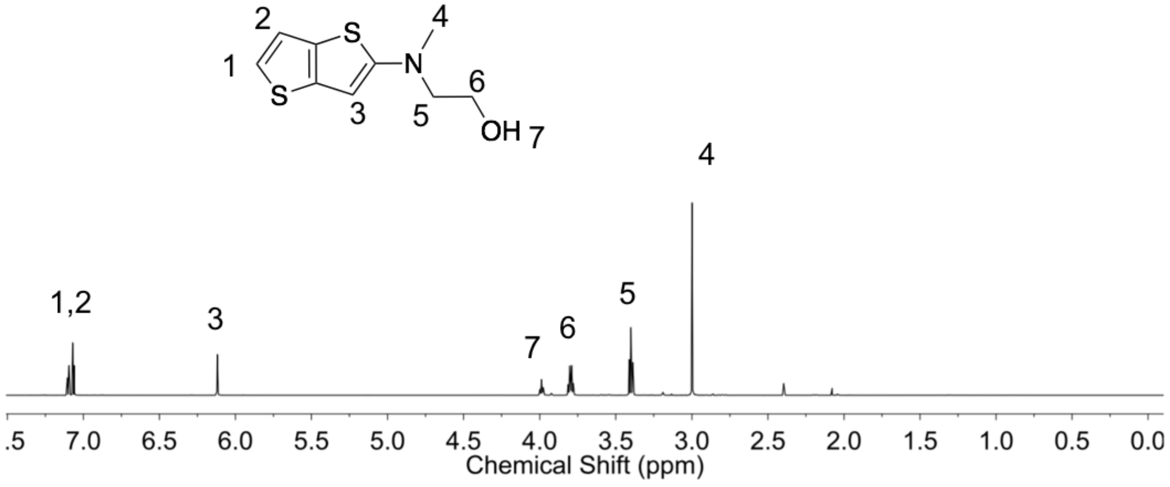

^{1}H NMR (500 MHz, Acetone-d_6) of **5**.

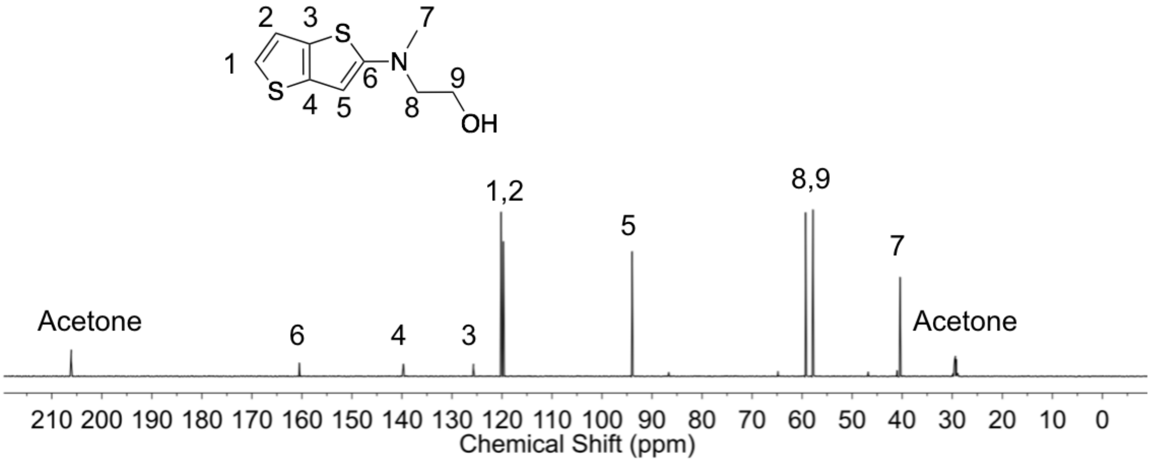

^{13}C NMR (126 MHz, Acetone-d_6) of **5**.

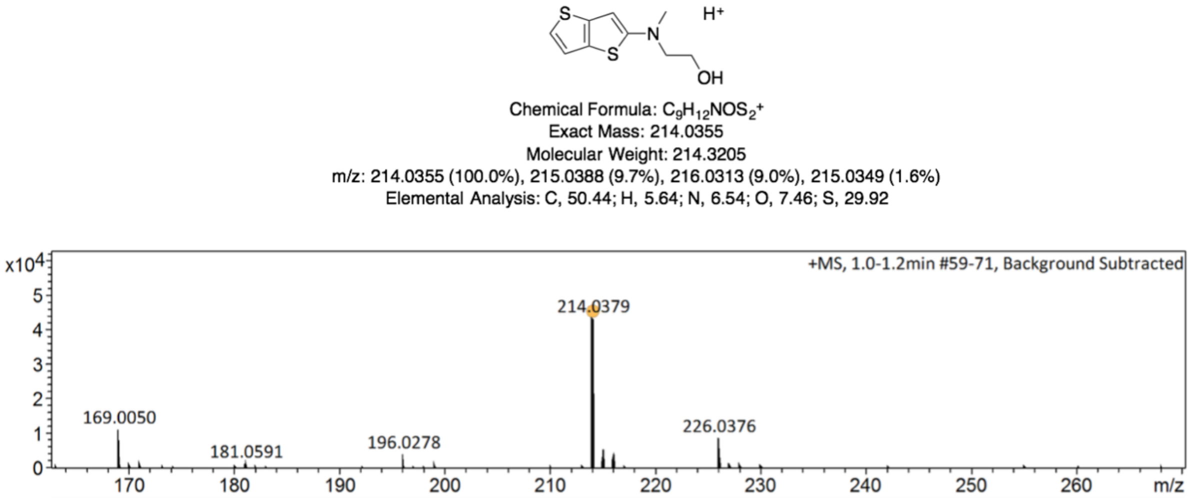

HRMS-ESI of **5**.

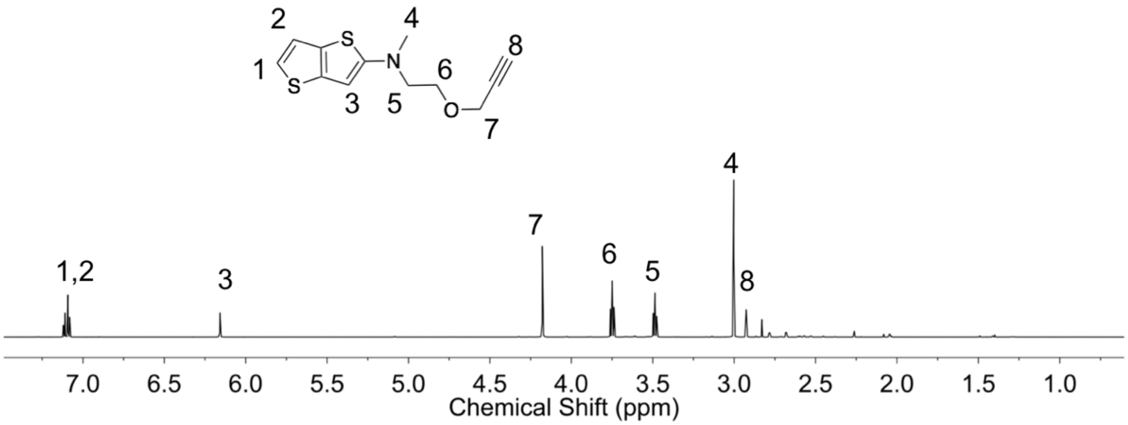

^{1}H NMR (500 MHz, Acetone-d_6) of **6**.

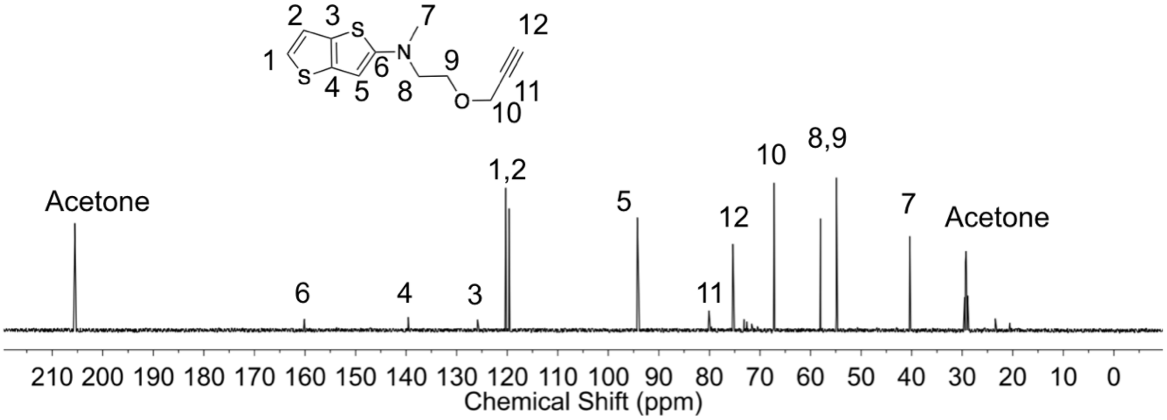

^{13}C NMR (126 MHz, Acetone-d_6) of **6**.

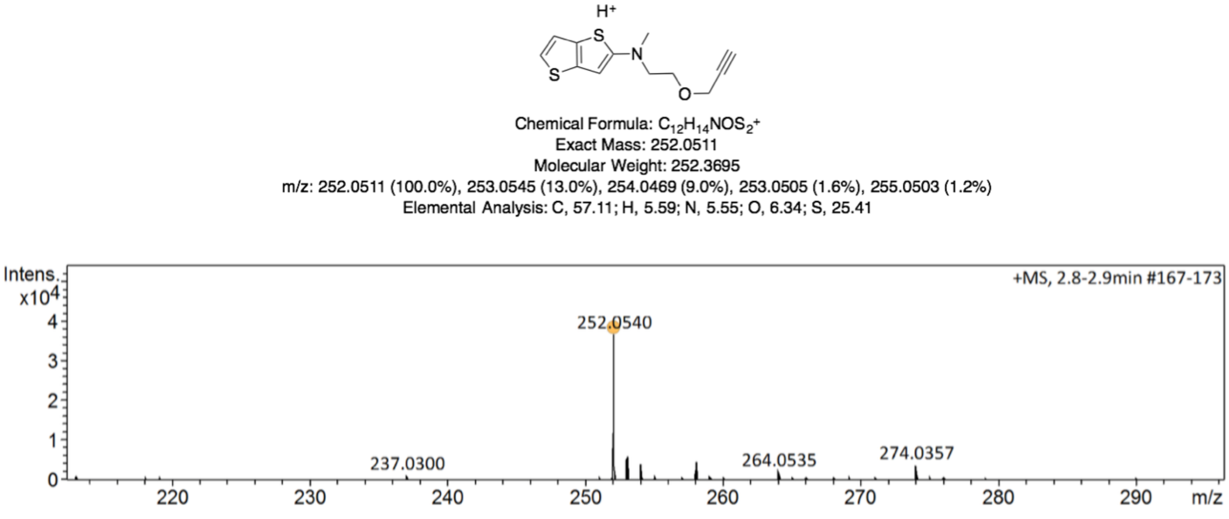

HRMS-ESI of **6**.

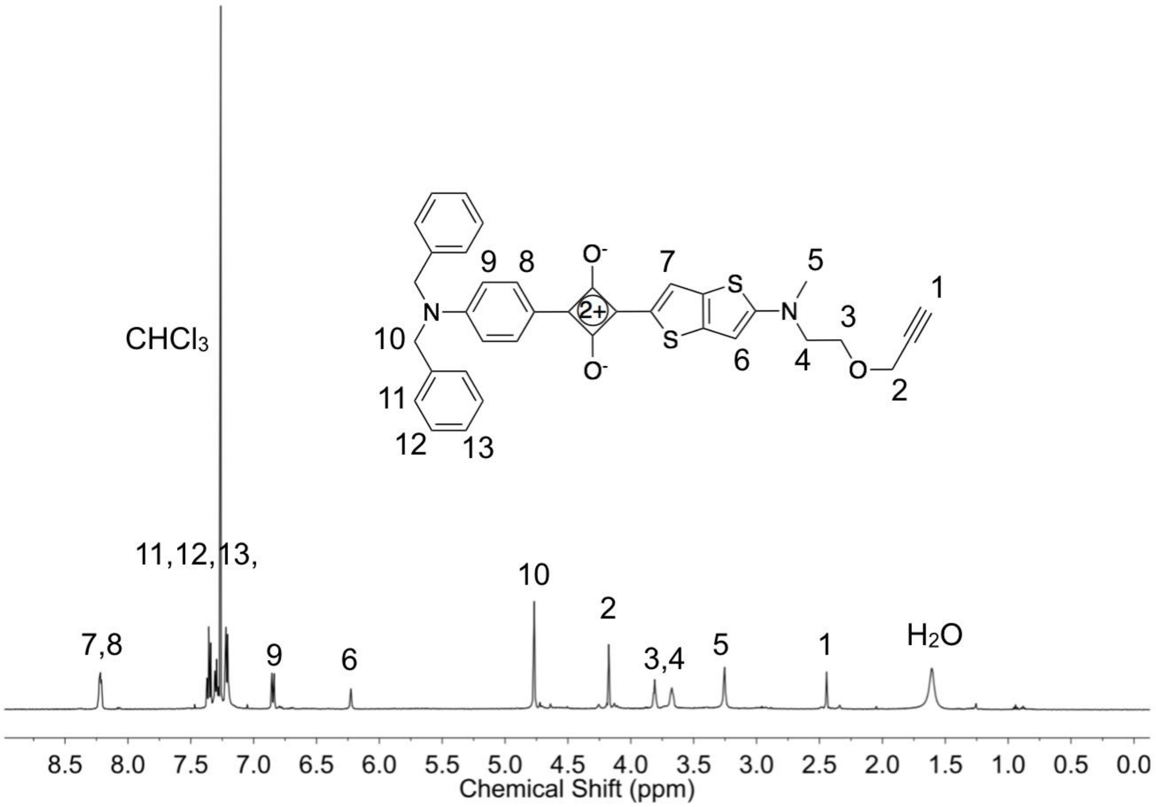

^{1}H NMR (500 MHz, $CDCl_3$) of **S2**.

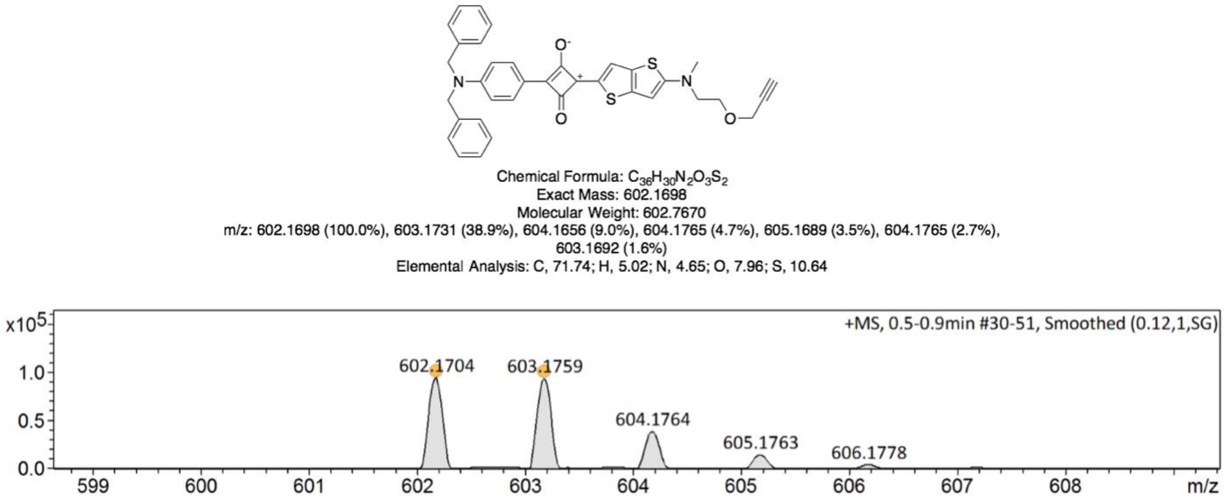

HRMS-ESI of **S2**.

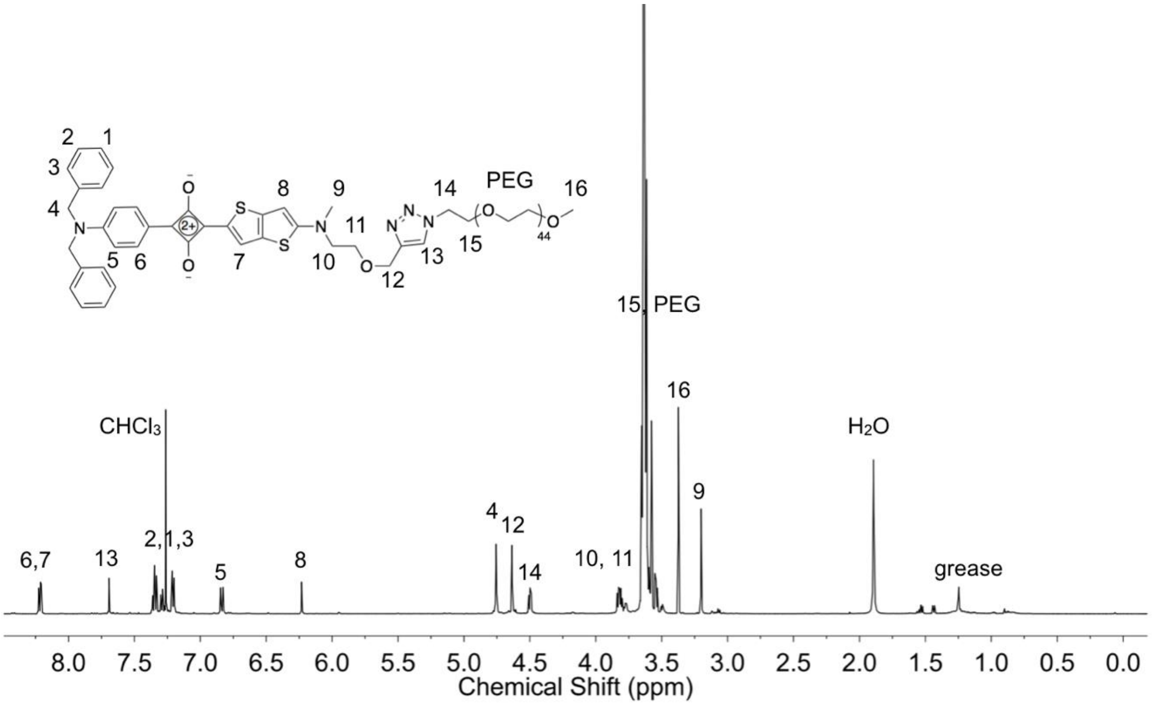

^{1}H NMR (500 MHz, $CDCl_3$) of **S2PEG**.

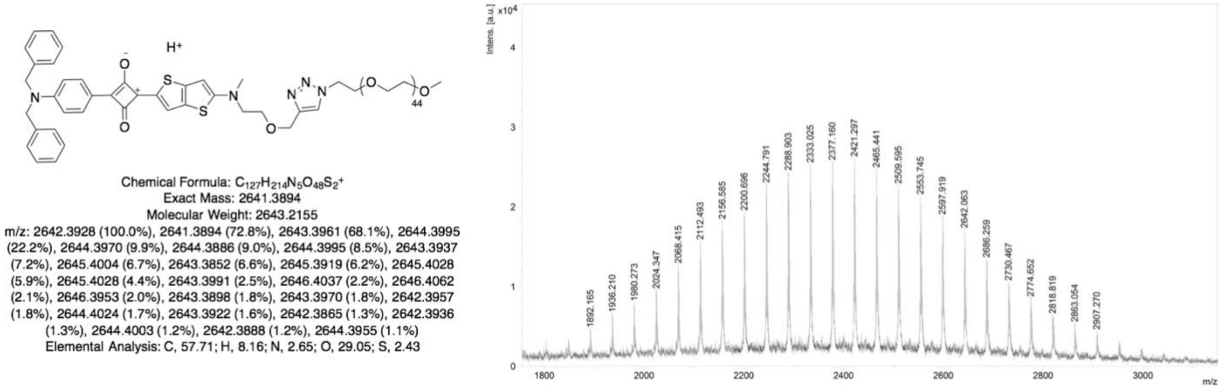

MS-MALDI (DHBA as matrix) of **S2PEG**. A set of peaks around 2421 reflect the polydispersity of the PEG_{45} chain.

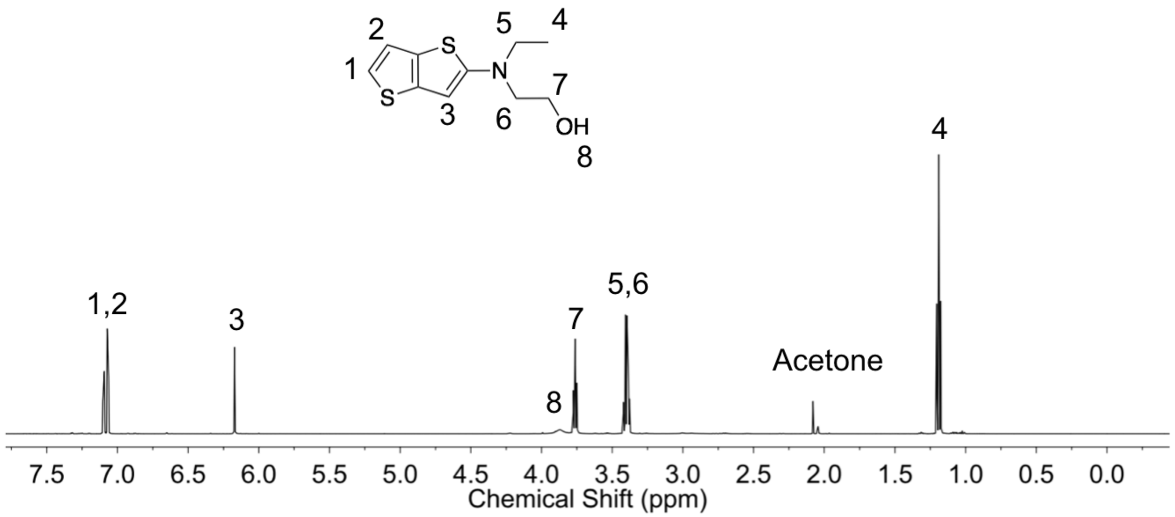

^{1}H NMR (500 MHz, Acetone-d_6) of **7**.

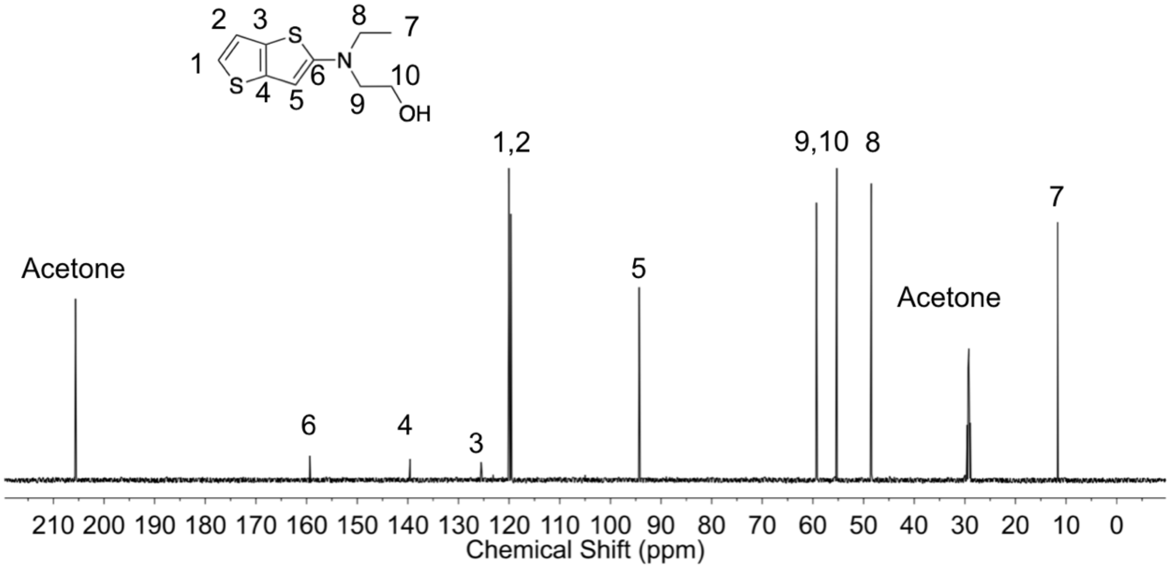

^{13}C NMR (126 MHz, Acetone-d_6) of **7**.

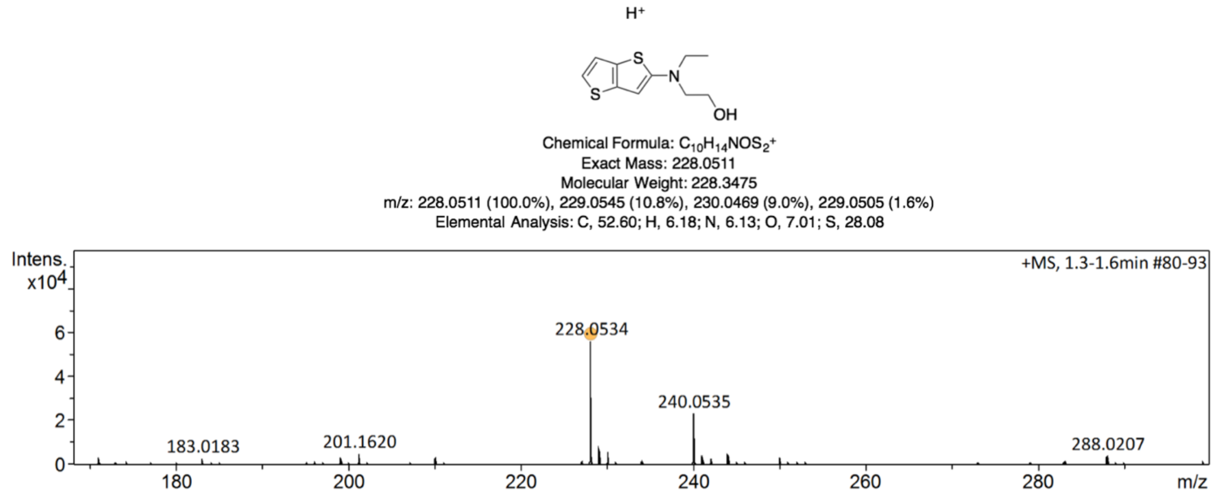

HRMS of **7**.

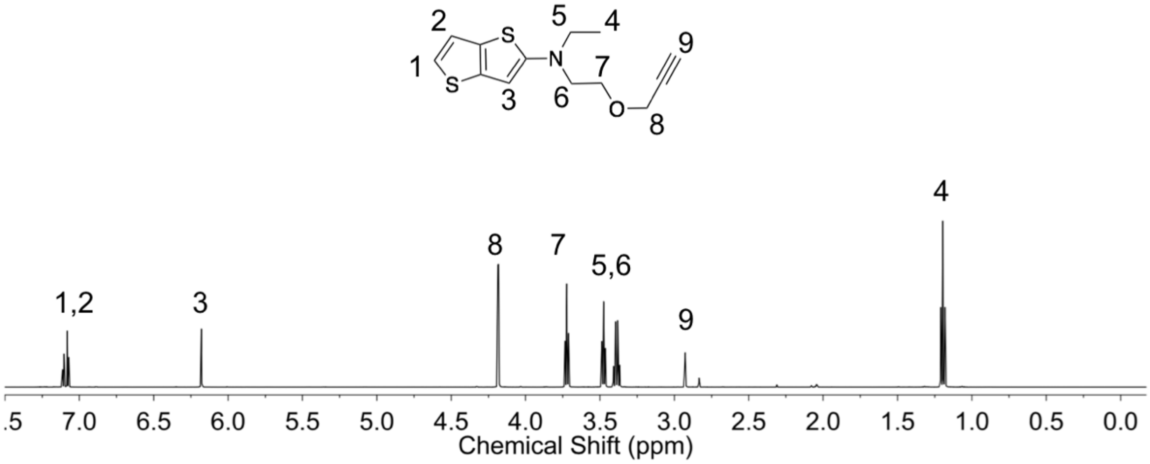

^{1}H NMR (500 MHz, Acetone-d_6) of **8**.

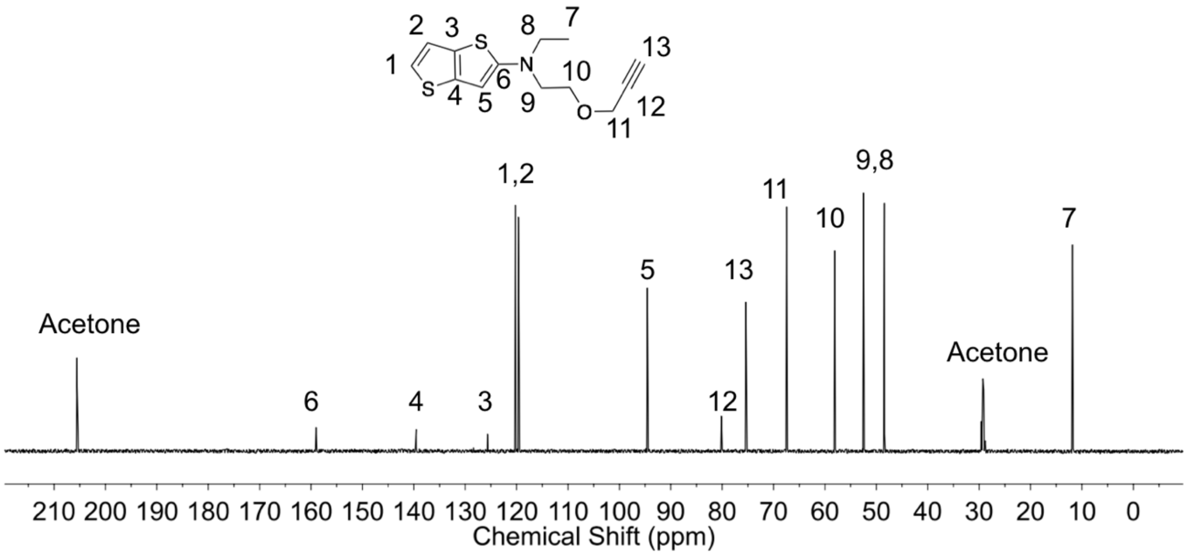

^{13}C NMR (126 MHz, Acetone-d_6) of **8**.

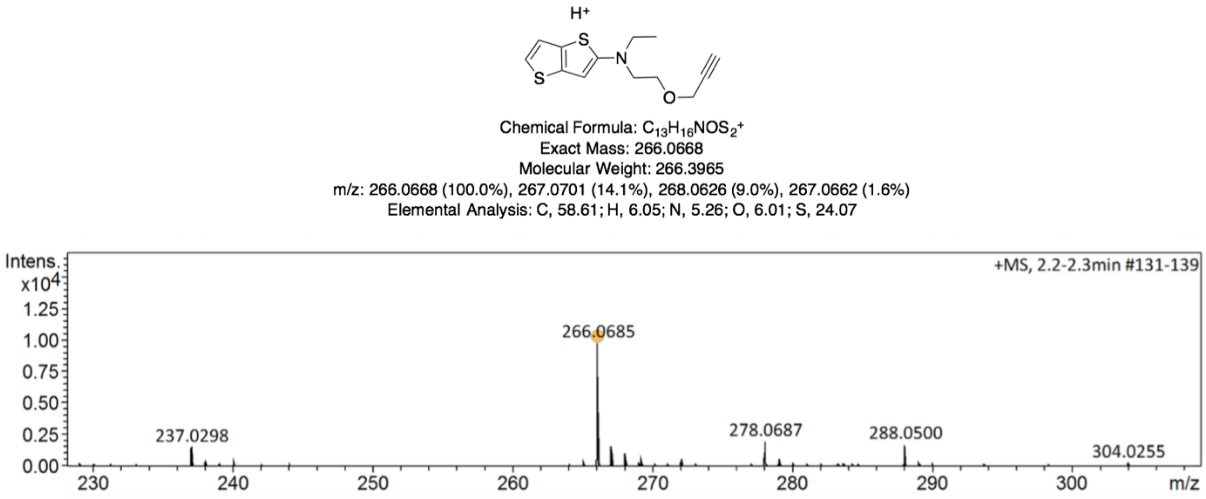

HRMS of **8**.

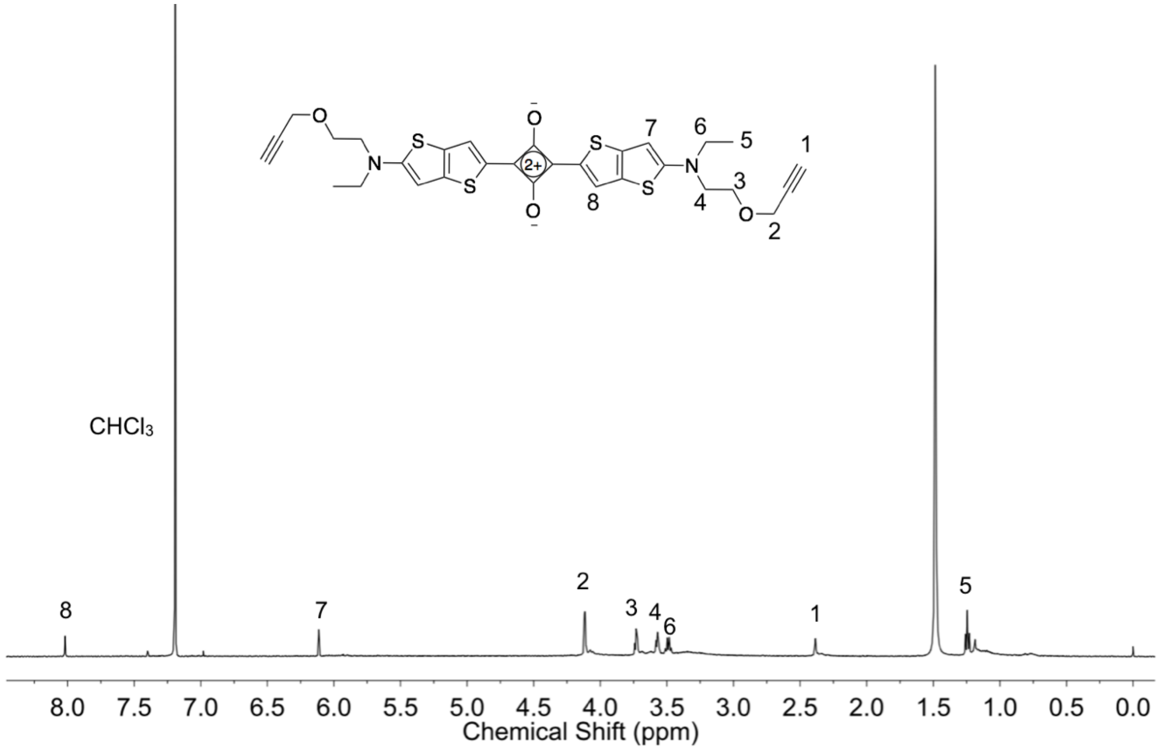

^{1}H NMR (500 MHz, $CDCl_3$) of **S3**.

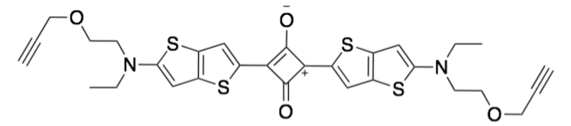

Chemical Formula: $C_{30}H_{28}N_2O_4S_4$
Exact Mass: 608.0932
Molecular Weight: 608.8040
m/z: 608.0932 (100.0%), 609.0965 (32.4%), 610.0890 (18.1%), 611.0923 (5.9%), 610.0999 (5.1%), 609.0926 (3.2%), 612.0848 (1.2%), 610.0959 (1.0%)
Elemental Analysis: C, 59.19; H, 4.64; N, 4.60; O, 10.51; S, 21.06

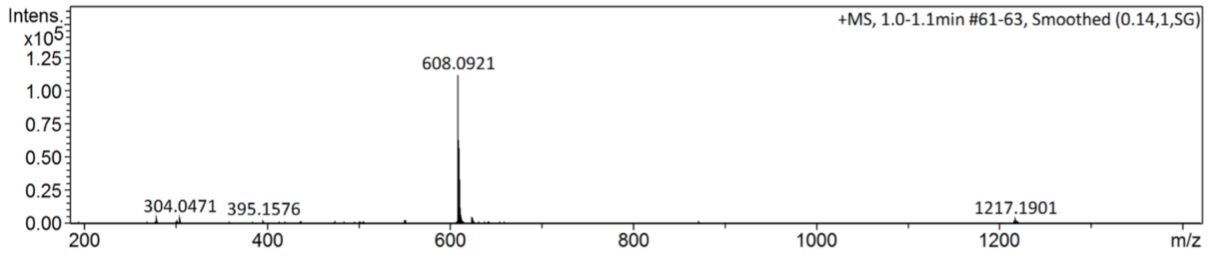

HRMS-ESI of **S3**.

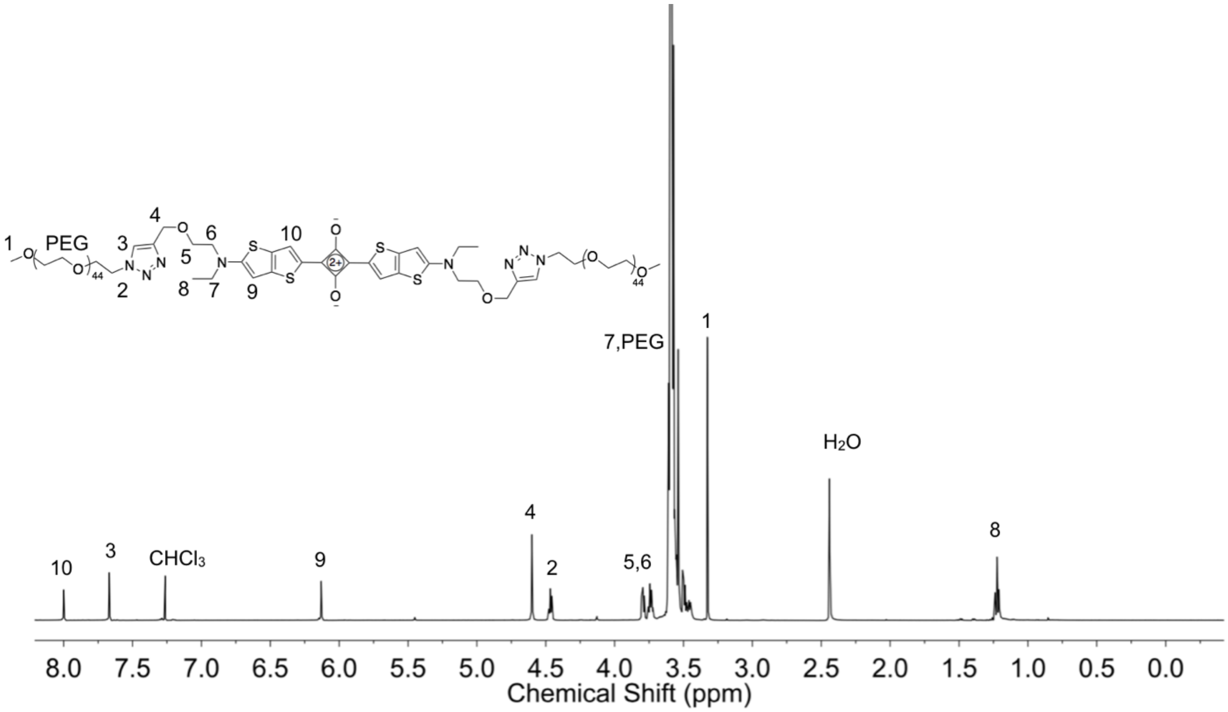

^{1}H NMR (500 MHz, $CDCl_3$) of **S3PEG**.

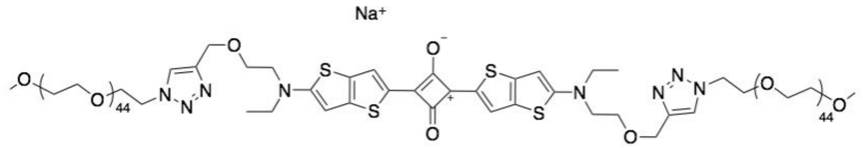

Chemical Formula: $C_{212}H_{394}N_8NaO_{94}S_4^+$
Exact Mass: 4707.5071
Molecular Weight: 4710.6752
m/z: 4709.5138 (100.0%), 4708.5105 (87.6%), 4710.5172 (75.7%), 4707.5071 (38.2%), 4711.5206 (28.4%), 4711.5181 (19.3%), 4712.5239 (18.1%), 4711.5096 (18.1%), 4710.5147 (16.9%), 4710.5063 (15.8%), 4711.5206 (14.0%), 4712.5130 (13.7%), 4712.5214 (12.2%), 4709.5114 (7.4%), 4709.5029 (6.9%), 4713.5248 (5.5%), 4713.5163 (5.1%), 4713.5139 (3.5%), 4714.5197 (3.3%), 4710.5132 (3.2%), 4713.5273 (3.2%), 4712.5105 (3.1%), 4710.5109 (3.0%), 4709.5099 (2.8%), 4709.5075 (2.6%), 4713.5163 (2.5%), 4711.5166 (2.4%), 4711.5142 (2.2%), 4714.5172 (2.2%), 4713.5273 (1.7%), 4711.5072 (1.3%), 4713.5054 (1.2%), 4708.5065 (1.2%), 4708.5042 (1.1%), 4712.5021 (1.1%)
Elemental Analysis: C, 54.05; H, 8.43; N, 2.38; Na, 0.49; O, 31.93; S, 2.72

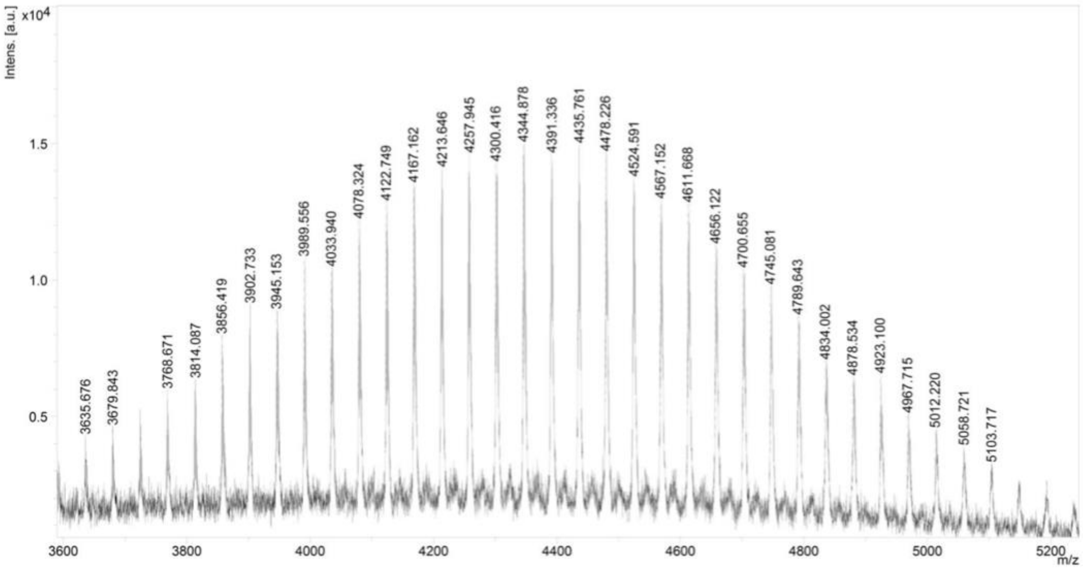

MS-MALDI (DHBA as matrix) of **S3PEG**. A set of peaks around 4391 reflect the polydispersity of the PEG_{45} chains.

B. Titration Data

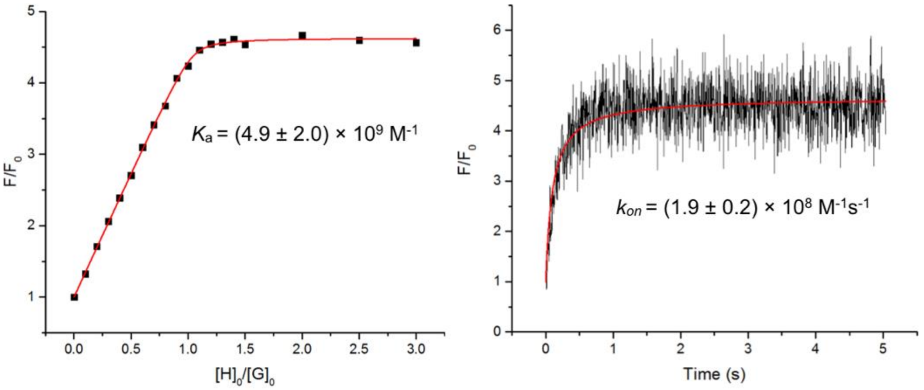

Figure S1. (*left*) Fluorescence (ex: 630 nm, em: 700 nm, slit 3 nm) titration isotherm for incremental addition of **M2** to a solution of **S1PEG** (250 nM) in water. (*right*) Threading kinetic profile generated by mixing equal molar concentration (50 nM each) of **S1PEG** and **M2** in a stopped flow device (ex: 630 nm, em: 700 nm, slit 3 nm). The red lines are computer fits of experimental data to 1:1 binding model or second order kinetic model, respectively.

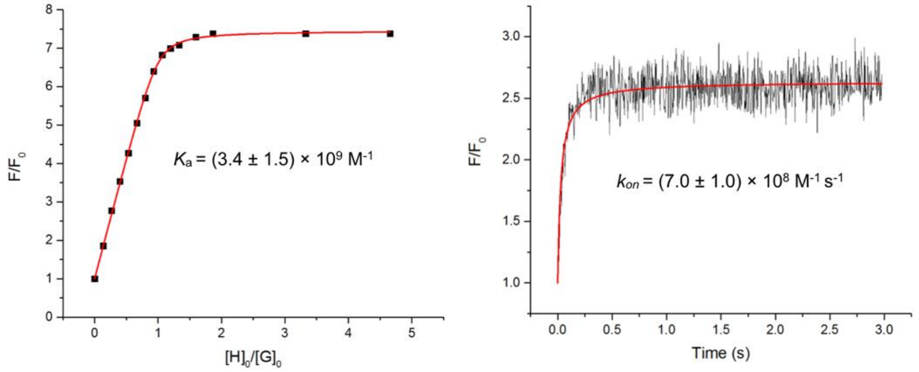

Figure S2. (*left*) Fluorescence (ex: 723 nm, em: 753 nm, slit 5 nm) titration isotherm for incremental addition of **M2** to a solution of **S2PEG** (250 nM) in water. (*right*) Threading kinetic profilegenerated by mixing equal molar concentration (50 nM each) of **S2PEG** and **M2** in a stopped flow device (ex: 723 nm, em: 753 nm, slit 3 nm). The red lines are computer fits of experimental data to 1:1 binding model or second order kinetic model, respectively.

C. Photostability Data

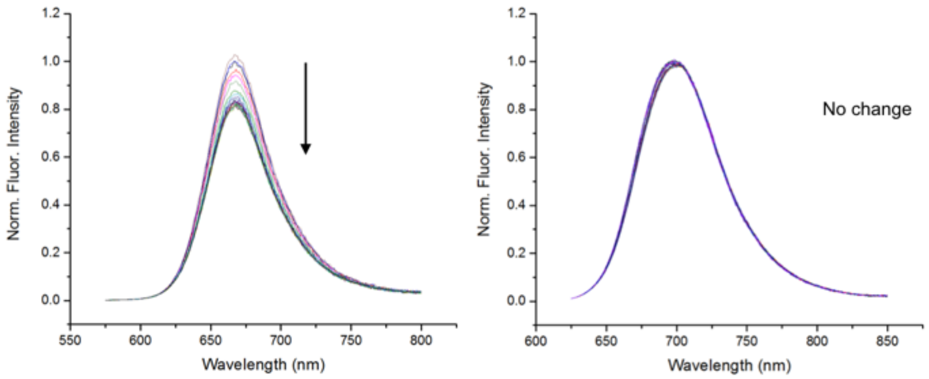

Figure S3. Photostability of free **S1PEG** (5 µM, *left*) and **M2** ⊃ **S1PEG** (5 µM, *right*) in H_2O with continuous irradiation at 550 nm over 15 h (fluorescence spectrum was collected every 30 min).

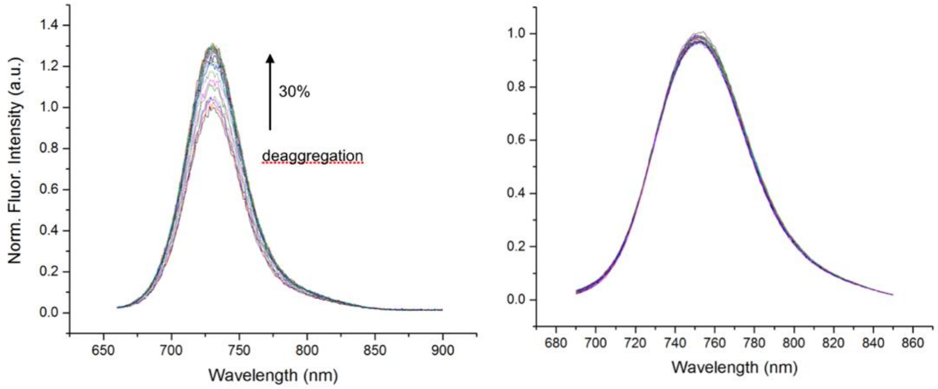

Figure S4. Photostability test of free **S2PEG** (2 µM, *left*) and **M2** ⊃ **S3PEG** (2 µM, *right*) in H_2O with continuous irradiation at 650 nm over 15 h (fluorescence spectrum was collected every 30 min). Note: slight increase in fluorescence over time for free **S2PEG** is attributed to slow deaggregation.

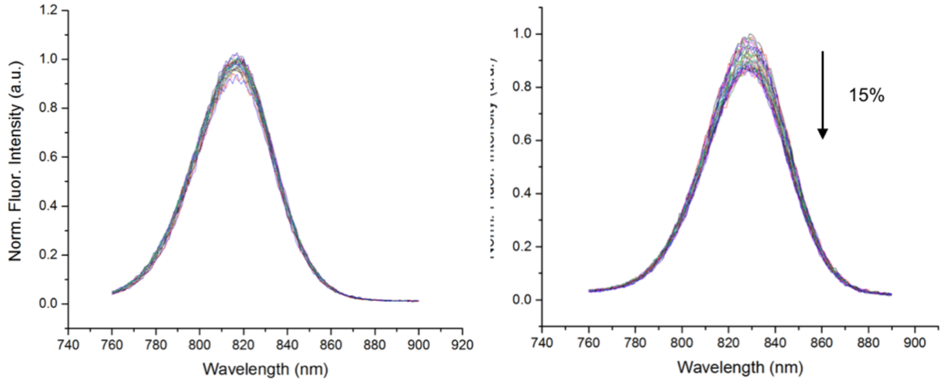

Figure S5. Photo stability test of the **S3PEG** (2 µM, *left*) and **M2** ⊃ **S3PEG** (2 µM, *right*) in H_2O with continuous irradiation at 750 nm over 15 h (fluorescence spectrum was collected every 30 min).

D. Chemical Stability Data

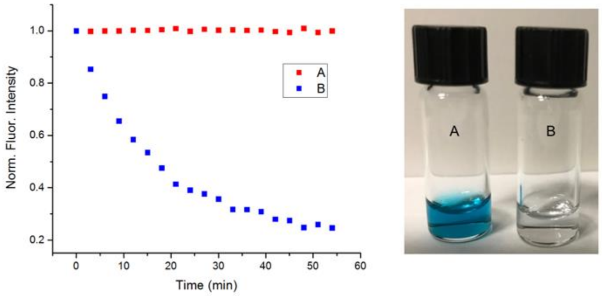

Figure S6. Chemical stability test of **S1PEG** and **M2** ⊃ **S1PEG**. (*left*) Change in fluorescent maxima band for solutions of (A) **M2** ⊃ **S1PEG** (5.0 µM) or (B) free **S1PEG** (5 µM), in the presence of excess nucleophile Na_2S (5 mM) in water at 20°C. (*right*) Photograph of samples containing, (A) **M2** ⊃ **S1PEG** (80 µM) or (B) free **S1PEG** (80 µM), after sitting in the presence of excess nucleophile Na_2S (100 mM) in water at 20 °C.

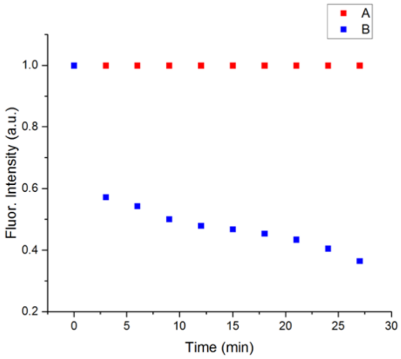

Figure S7. Chemical stability test of **S2PEG** and **M2** ⊃ **S2PEG** by monitoring the change in fluorescent maxima band for solutions of (A) **M2** ⊃ **S2PEG** (5.0 µM) or (B) free **S2PEG** (5 µM) over time in the presence of excess nucleophile Na_2S (5 mM) in water at 20 °C.

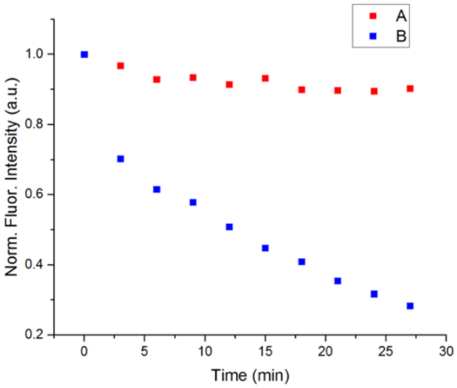

Figure S8. Chemical stability test of **S3PEG** and **M2** ⊃ **S3PEG** by monitoring change in fluorescent maxima band for solutions of (A) **M2** ⊃ **S3PEG** (1.0 µM) or (B) free **S3PEG** (1 µM), in the presence of excess nucleophile Na_2S (1 mM) in water at 20 °C.

E. Fluorescence Quantum Yields

Table S1. Integrated fluorescent and quantum yield of **ICG**, **S3PEG**, and **M2** ⊃ **S3PEG** in H_2O.

	**ICG**	**S3PEG**	**M2 ⊃ S3PEG**
Integrated fluorescence	2.2×10^6	2.9×10^6	4.4×10^6
Quantum yield %	5.3	7.0	10.6

Table S2. Photophysical properties of squaraines and their complexes with **M1** in $CHCl_3$.

	**S1**	**M1⊃S1**	**S2**	**M1⊃S2**	**S3**	**M1⊃S3**
Abs (nm)	627	650	692	717	767	783
Em (nm)	651	685	715	749	792	820
logε ($M^{-1}cm^{-1}$)	5.39	4.68	5.48	4.84	5.59	5.02
